# Supplementary material for: Prediction of liver disease in patients whose liver function tests have been checked in primary care: model development and validation using population-based observational cohorts
Source: BMJ Open. 2014 Jun 2;4(6):e004837. doi: 10.1136/bmjopen-2014-004837 (PMC4054629; doi:10.1136/bmjopen-2014-004837)
Supplement: Author's manuscript [file bmjopen-2014-004837.draft_revisions.pdf]

Prediction of liver disease in patients whose liver function tests have been checked in primary care: model development and validation using population-based observational cohorts

Journal:	*BMJ Open*
Manuscript ID:	bmjopen-2014-004837
Article Type:	Research
Date Submitted by the Author:	10-Jan-2014

Complete List of Authors:	McLernon, David; University of Aberdeen, Division of Applied Health Sciences<br>Donnan, Peter; University of Dundee, Dundee Epidemiology and Biostatistics Unit<br>Sullivan, Frank; University of Dundee, Population Health Sciences<br>Roderick, Paul; University of Southampton, Faculty of Medicine<br>Rosenberg, William; UCL,<br>Ryder, Stephen; Nottingham<br>Dillon, John; University of Dundee, Ninewells Hospital and Medical School

**Primary Subject Heading**:	General practice / Family practice
Secondary Subject Heading:	Gastroenterology and hepatology, Epidemiology
Keywords:	PRIMARY CARE, Hepatology < INTERNAL MEDICINE, EPIDEMIOLOGY

SCHOLARONE™ Manuscripts

For peer review only

Prediction of liver disease in patients whose liver function tests have been checked in primary care: model development and validation using population-based observational cohorts

David J McLernon[1*]
* Corresponding author
Email: d.mclernon@abdn.ac.uk

Peter T Donnan[2]
Email: p.t.donnan@dundee.ac.uk

Frank M Sullivan[3]
Email: f.m.sullivan@dundee.ac.uk

Paul Roderick[4]
Email: pjr@soton.ac.uk

William M Rosenberg[5]
Email: w.rosenberg@ucl.ac.uk

Steve D Ryder[6]
Email: Stephen.Ryder@nuh.nhs.uk

John F Dillon[7]
Email: j.dillon@nhs.net

[1]Medical Statistics Team, Division of Applied Health Sciences, College of Life Sciences and Medicine, University of Aberdeen, UK

[2]Dundee Epidemiology and Biostatistics Unit, Division of Population Health Sciences, Medical Research Institute, University of Dundee, Dundee, UK

[3]Division of Population Health Sciences, Medical Research Institute, University of Dundee, Dundee, UK

[4]Academic Unit of Primary Care and Population Sciences, Faculty of Medicine, University of Southampton, Southampton, UK

[5]Centre for Hepatology, Division of Medicine and ULCH-UCL NIHR Biomedical Research Centre, University College London, London, UK

[6]Department of Gastroenterology, Nottingham University Hospitals NHS Trust and Biomedical Research Unit, Nottingham, UK

[7]Medical Research Institute, University of Dundee, Dundee, UK.

For peer review only

Abstract

Objective: To derive and validate a clinical prediction model to estimate the risk of liver disease diagnosis following an abnormal liver function test (LFT) result and to convert the model to a simplified scoring tool for use in primary care.

Design: Population-based observational cohort study of patients in Tayside Scotland identified as having their LFTs performed in primary care and followed for two years. Biochemistry data was linked to secondary care, prescriptions and mortality data to ascertain baseline characteristics of the derivation cohort. A separate validation cohort was obtained from 19 general practices across Scotland to externally validate the final model.

Setting: Primary care, Tayside, Scotland

Participants: Derivation cohort: LFT results from 310 511 patients. After exclusions (including: patients under 16 years, patients having initial abnormal LFTs measured in secondary care, bilirubin >35 µmol/L, complications within 6 weeks and history of a liver condition) derivation cohort contained 95 977 patients with no clinically apparent liver condition. Validation cohort: After exclusions this cohort contained 11 653 patients.

Primary and secondary outcome measures: Diagnosis of a liver condition within two years.

Results: From the derivation cohort (n=95 977), 481 (0.5%) were diagnosed with a liver diseaseThe model showed good discrimination in both cohorts (C-statistic=0.78). A predicted risk <5% had good ability to 'rule out' liver disease (specificity=99.4%, negative predictive value=99.5%).

Conclusions: This study successfully developed and validated a clinical prediction model and subsequent scoring tool, the Algorithm for Liver Function Investigations (ALFI), that can predict liver disease risk in patients, with no clinically obvious liver disease, who had their initial LFTs taken in primary care. GPs who use ALFI to support their decision-making process can reassure patients at low risk. Clinical review of low risk patients instead of

For peer review only

further investigations has the potential to reduce iatrogenic disease and save health service costs.

Strengths and limitations of this study

- Our study has successfully developed and validated the first risk prediction model and subsequent user-friendly scoring tool, the Algorithm for Liver Function Investigations (ALFI), for liver condition diagnosis in patients with no obvious liver condition at the time of incident liver function testing in primary care.
- This model can be used to facilitate GP decision making with regards to referral to secondary care.
- GGT was found to be a powerful predictor of liver disease, over and above alkaline phosphatase and transaminase. Since some laboratories do not routinely test for GGT its use should be re-evaluated.
- The observational data lacked some potential predictors of liver disease e.g. alcohol intake and body mass index. However, other available predictors such as LFTs and deprivation may act as surrogate markers for such factors.

Background

For peer review only

GPs commonly request liver function tests (LFTs) but the results rarely identify cases of liver disease [1]. Abnormal liver function tests (LFTs) can be indicators of many different diseases besides those of the liver, such as metastatic malignancy, congestive heart failure and systemic inflammatory conditions such as viral infections [2-5]. It can therefore be difficult for a GP to identify a specific liver disease in patients with abnormal LFTs particularly in those who do not have pathognomonic signs or symptoms. This leads to variation in approaches to further investigation with some patients being inadequately investigated and others at low risk of disease having invasive tests with attendant morbidity or mortality [6]. The size of this problem is huge with 22% of patients having their initial panel of LFTs performed in general practice having at least one abnormality in Tayside, Scotland [7]. Referral of all these patients to secondary care would place a large burden on health care system resources, so guidance is needed to ensure that care is focussed on those most in need.

The primary care management of these types of patients might be facilitated through the use of a clinical prediction model which estimates the risk of a specific outcome adjusted for patient characteristics [8]. These models, such as the Framingham cardiovascular disease risk score [9], and FRAX for osteoporotic fracture are regularly used in primary care [10]. It is important that clinical prediction models are assessed for their predictive ability, preferably on an external cohort [11].

This population-based historical cohort study followed-up two separate cohorts of patients living in Scotland with no clinically recognised liver disease who initially had LFTs undertaken in primary care [12]. The aims of the study were:

For peer review only

1. To derive a clinical prediction model using a cohort of patients from Tayside that would estimate the risk of a liver disease over the two years following LFTs taken in primary care;
2. To validate this model geographically and temporally using a different cohort of patients from 19 general practices in Scotland;
3. To convert this model into a user-friendly clinical scoring tool that will allow the GP to easily calculate the risk of liver disease diagnosis at 6 months and 2 years.

Methods

Separate populations were used to develop the prognostic model (derivation cohort) and then validate it (validation cohort).

Derivation cohort

The study population was initially derived from a laboratory database which contains all electronically available LFT results from patients within Tayside during the fifteen year period from January 1989 to December 2003 linked to hospital admission and GP prescribing data [12]. LFTs included bilirubin, albumin, alkaline phosphatase (ALP), gamma-glutamyltransferase (GGT), alanine transaminase and aspartate aminotransferase. Since many laboratories only measure either alanine transaminase or aspartate aminotransferase these two tests were combined as one test and are referred to as transaminases throughout.

Patients aged 16 and above with no obvious or reported clinical signs of a liver disease upon presentation to their GP, with at least 2 different LFTs requested from the index appointment, between 1989 and 2003 were eligible for inclusion. The following exclusion criteria ensured

For peer review only

that the study population of patients had no clinically recognised liver disease at presentation in primary care:

- Bilirubin greater than 35 µmol/L at baseline, suggesting jaundice.
- Diagnosis of ascites, encephalopathy, varices or portal hypertension within 6 weeks of their first LFTs.
- History of any liver disease before baseline.

Databases

The databases relevant to this study are described briefly in Box 1 and in further detail elsewhere [12]. In Scotland, all individuals registered with a GP have a unique identifier, the Community Health Index (CHI) [14]. This number is used for all health encounters and is contained within all of the databases used in this study. Since it contains person identifiable information, the CHI was mapped to a project specific pseudonymous ten digit code by the data protection officer at the Health Informatics Centre, University of Dundee, before removing the CHI and releasing the data to the researcher. All of the electronic databases were electronically record-linked deterministically using this project specific pseudonymous code.

Ethics statement

Ethical approval was obtained from the Tayside Committee for Medical Research Ethics in February 2005 (Ref No. 04/S1401/199). Written informed consent from patients was waived by the Tayside Committee for Medical Research Ethics because the databases were anonymised so that no patient identifiable information was accessible. The databases used in this study (see Box 1) covered the entire study period and were used in accordance with

For peer review only

procedures approved under the Caldicott Guardian and the Data Protection Act UK (1998) in line with the European directive of 1995.

Baseline characteristics

As well as the five analysed LFTs, baseline characteristics included age, gender, deprivation [13], comorbidities (including cancer, diabetes, ischaemic heart disease (IHD), stroke, renal disease, respiratory disease and biliary disease), diagnosed alcohol and drug dependency, methadone use, and the use of statins, non-steroidal anti-inflammatory drugs (NSAIDs) or antibiotics in the three months before LFTs. Since patients with bilirubin >35 μmol/L were excluded, bilirubin was categorised into normal and mildly raised (<18 and 18-35 μmol/L respectively for males; <16 and 16-35 μmol/L respectively for females).

Outcome

The primary outcome for this study was liver disease diagnosis during the two years following the initial analysis of LFTs in primary care. Liver diseases were identified from the ELDIT database (Box 1), described in further detail elsewhere [18]. A detailed table of the liver diseases included and their source database is presented in Appendix 1 (available online).

Model derivation

Survival analysis was conducted using a parametric regression model to estimate the risk of liver disease within two years. The starting point was taken as the date of the initial LFT test and the endpoint for each patient was whichever one of the following events came first: end of follow-up (i.e. two years later), death, end of study period (i.e. 31st December 2003), date

For peer review only

of emigration, or liver disease diagnosed. All patients whose endpoint was not liver disease diagnosis were censored in the model.

The model was adjusted for the potential predictors at baseline. A manual stepwise technique was used to arrive at a model that contained only significant predictive characteristics. Clinically important two-way predictor interactions were also investigated and were included in the final model if they were statistically significant. A multiple imputation technique was conducted to impute missing values for the LFTs [19]. The integrated discrimination index (IDI) was used to measure the improvement in the final model for each individual covariate [20]. The IDI for a covariate is essentially the difference between the proportion of variance explained by the full model (i.e. adjusted for all covariates) and the model without the covariate of interest. The sensitivity, specificity, PPV, and negative predictive value (NPV) were also calculated for different risk cut-offs, accounting for censoring [20].

Model assessment

The final model was assessed for its predictive ability. The C-statistic was used as a measure of discrimination [21]. Discrimination assesses the model's ability to correctly distinguish between patients who have liver disease (for whom the model assigns a high risk) and do not have liver disease (for whom the model assigns a low risk). The model's predicted probabilities were assessed for accuracy using calibration plots (further information in Appendix 2) [22]. A calibration slope test was conducted to test for overfitting of the model [22]. This was done by fitting the linear predictor of the final model in a model by itself and testing its slope. A significant deviation from one signifies overfitting.

Model validation

For peer review only

A separate cohort of patients was obtained from the Primary Care Clinical Informatics Unit (PCCIU), University of Aberdeen [23], and was used to externally validate the final model. The validation cohort contained all patients registered with 19 practices from across Scotland out with Tayside. The practices were participating in the Practice Team Information project operated by the Information Services Division of the National Health Service National Services Scotland, and contributed data to PCCIU. The patient population within the PCCIU database is broadly representative of the Scottish population, with respect to age, sex, and social deprivation [24]. The validation cohort contained patients having their initial LFTs measured in primary care between January 2004 and August 2008. All eligible patients had to have test results for ALP, bilirubin, albumin, and transaminase. All baseline characteristics and outcome data obtained for the derivation cohort were also obtained for the validation cohort. The same exclusion criteria listed above was also applied to the validation cohort.

The parameter estimates of the final model from the derivation cohort were applied to the validation cohort to calculate predicted probabilities of liver disease. The same model assessment methods of discrimination and calibration as detailed above were then performed.

Clinical scoring tool

A points-based scoring tool was created from a simpler version of the final model for potential use in primary care [25]. Continuous predictors from the model were dichotomised into clinically and statistically sensible categories to create the tool. Mid-points of the categories were used to estimate the risk of liver disease for each category.

Analyses were performed using SAS (v9.3) (SAS Institute, Cary, North Carolina).

For peer review only

Results

Baseline characteristics

Before applying the exclusion criteria LFTs were extracted for 310 511 patients. After excluding patients under 16 years old, non-Tayside residents and those who initial LFTs were measured in secondary care, 99 165 patients remained. After excluding those with clinically recognised liver disease at baseline the derivation cohort contained 95 977 patients with incident initial LFTs taken in primary care and with no obvious liver disease. There were more females (57.9%) than males (42.1%), and the median (interquartile range) age was 54.6 (39.2 – 68.8) years (Table 1). The most frequent co-morbidity was IHD (5.6%), followed by cancer (3.8%).

Only 8 388 (8.7%) patients had all five LFTs. The percentage of complete data for each LFT was as follows: ALP (99.2%), albumin (99.2%), bilirubin (93.6%), transaminases (76.5%), and GGT (10.9%). There were more males with complete data (i.e. having all five LFTs) than females (54.6% versus 45.4%). The group with complete data were also more deprived and contained more alcohol dependent patients than the incomplete data group.

Liver disease diagnosis

A total of 481 patients (0.5%) were diagnosed with a liver disease during the two year follow-up period. Of these, 339 (70.5%) had at least one abnormal LFT (out of 22 673). Cirrhosis was the most frequently diagnosed condition (n=75, 15.6%), and in 19 of these the cause was recorded as alcohol related liver disease. PBC was diagnosed in 73 patients but only as definite in 13 (probable, n=37; possible n=23). The next most frequent diagnoses were Hepatitis C (n=68), alcoholic related liver disease (n=68), Hepatitis B (n=40), hepatocellular carcinoma (n=34), autoimmune hepatitis (n=29) and fatty liver disease (n=28).

For peer review only - http://bmjopen.bmj.com/site/about/guidelines.xhtml

Prediction of a liver disease

The following baseline characteristics were significant predictors of liver disease diagnosis: increasing GGT, decreasing albumin, alcohol dependency, being female, increasing ALP, living in a deprived area and younger age (Table 2). There were also significant interactions between albumin and transaminase, and albumin and methadone use. NSAID use, antibiotic use, drug dependency, and all comorbidity history indicators were not significantly predictive of a liver disease. The IDI statistic showed that albumin explained the greatest percentage of variance in the model followed by GGT.

The discriminatory ability of the model was good with a C-statistic of 0.78 (95% CI 0.73, 0.83). For the model without LFTs the C-statistic was 0.59 (95% CI 0.52, 0.65), and proves that LFTs add considerable discriminatory ability to the model. The calibration curve (Appendix 2) shows reasonable agreement between the predicted and actual probabilities. However, the calibration slope deviated significantly from 1 (slope = 1.129, 95% CI 1.069 to 1.189) suggesting some evidence of overfitting. However, with large sample sizes the chance of tests being statistically significant increase and the magnitude of the difference of the slope from 1 is not particularly large.

The sensitivity, specificity, PPV and NPV for different cut-offs of predicted risk of liver disease diagnosis along with the receiver operating characteristic curves for the final model applied to the derivation and external validation cohorts are displayed in Appendix 3. A cut-off greater than or equal to the 75th percentile, 0.43%, had sensitivity and specificity of approximately 75% each. The NPV was very high at almost 100% for all cut-offs which is useful when trying to avoid unnecessary tests. The PPV was low since liver disease diagnosis

For peer review only

was relatively rare. These performance measures were similar for both derivation and validation cohorts.

Validation

After exclusions, the external cohort contained 11 653 patients with incident initial LFTs taken in primary care. The proportion of males and females were reasonably similar to the derivation cohort (45.2% versus 42.1% males), but the external cohort was older, more deprived and had a greater proportion of co-morbidities and medications (Table 1). However, the median LFTs were similar between the two cohorts. GGT was missing for 4178 (35.9%) patients and was imputed using the same method as for the derivation cohort. A total of 57 patients (0.5%) were diagnosed with a liver disease within two years.

The C-statistic for the final model parameter estimates applied to the external cohort was 0.78 (95% CI 0.72 to 0.84). The calibration plot (Appendix 2) showed some visible evidence of over-fitting for the highest risk group, however the calibration slope showed no significant deviation from 1 (slope = 0.871 (95% CI 0.696 to 1.047); p=0.15).

Predicted probabilities

The final model and how to calculate the risk of liver disease directly from it is presented in Appendix 4. Figure 1 shows the risk of diagnosis over two years for the average risk patient and two example patients with different characteristics as described in the legend.

Clinical scoring tool

A user friendly paper-based scoring tool was developed from a slightly simpler version of the final model with the albumin and transaminase interaction term removed (Figure 2). The

For peer review only

scores for each of the risk factors add up to a total score that corresponds to the risk of liver disease. The baseline case for the tool was a 45 year old female, living in an affluent area, who was not alcohol or methadone dependent with transaminase=11.5U/L, ALP=46 U/L, GGT=17 U/L and albumin=48g/L. Her score of zero corresponded to a liver disease risk of 0.03%. As an example, a 60 year old male living in a deprived area who is alcohol dependent, but not a methadone user, with transaminase=70U/L, ALP=130U/L, GGT=45U/L and albumin=38 has a score of 29 which corresponds to a liver disease risk within two years of 4.1%. For comparison, when this patient's characteristics were entered into the full model in appendix 4 their risk was 3.8%.

Discussion

Summary

A user-friendly clinical scoring tool has been developed that can estimate the risk of liver disease diagnosis at six months and two years in patients who had their initial LFTs measured in primary care. The tool is based on a clinical prediction model which was derived and validated using large population databases. This model adds morbidity and a longer duration to a previous algorithm that predicts one year mortality as part of our Algorithms for Liver Function Investigations (ALFI) [26].

Strengths and limitations

This is the first large-scale population-based analysis of LFTs with complete determination of liver disease outcome. ALFI has been derived from unselected "real-world" observations in a geographically defined population: an approach recommended by the National Institutes of Health [27]. A weakness of the electronic databases was the lack of some potential predictors of liver disease e.g. alcohol intake and body mass index, which the GP can readily ascertain

For peer review only

to guide decisions. However, predictors in the model such as LFTs and deprivation may act as surrogate markers for such factors. Whilst we have no data on the clinical indications for LFTs being measured, we were able to identify patients with a history of major co-morbidities since 1980, including cancer, diabetes and IHD using hospital admission records and population registers, and patients who were prescribed statins, NSAIDs, and antibiotics. Using the predictors we did have, the model was proven to have good ability to predict liver disease. There will have been underascertainment of some liver diseases; particularly those diagnosed in primary care, such as mild alcohol related liver disease and the steatotic end of the spectrum of non-alcoholic fatty liver disease. Patients with these diseases are most likely to be dealt with by being advised to lose weight or reduce alcohol intake, and only those who developed further problems and who were referred would be captured on the database.

The aim of the study was to combine known significant predictors of liver disease into one source of prognostic information that could provide the GP with an individualised estimate of the predicted risk of liver disease at the point where the patient has had their initial LFTs measured. If, at that point, the patient has a high risk of liver disease based on this model then the GP can also use other contextual information and their own clinical judgement to make a decision as to whether to monitor or refer the patient to a liver clinic according to national or local protocols.

External validation

The validation cohort came from 19 general practices across nine different regions of Scotland during a different time period than the derivation cohort. Good discrimination and calibration demonstrated that ALFI is transportable to different geographical and temporal populations. The C-statistic for discriminatory ability was 0.78 which is comparable to that

For peer review only

found in the validation of the Framingham equation on different cohorts ranging from 0.63 to 0.83 [28]. The external cohort also contained more deprived and comorbid patients than the derivation cohort proving that ALFI is also robust to changes in baseline characteristics.

Implications for practice and research

At a cut-off of 0.43% (the 75th percentile) sensitivity and specificity were similar with values of 74.1% and 75.2% respectively. However, the PPV for this cut-off was poor at 1.5%. The specificity and NPV of the model are very high even for reasonably small cut-offs of risk meaning that the model is good at ruling out risk of liver disease within two years [29]. For example, a cut-off of 5% (specificity 98.9% and NPV 99.6%) could be used to rule out patients with lower risk in combination with clinical judgement. Such an approach could avoid unnecessary further investigations. However, the long timeframe of the development of many liver diseases means that general practitioners should also counsel over liver disease risk e.g. alcohol misuse. The next step is to quantify the clinical usefulness of ALFI and evaluate it as a complex intervention [30].

GGT was missing for a large proportion of patients in the derivation cohort but much less so in the validation cohort. Since only albumin explained more variation in the model, GGT should be considered just as important as other LFTs in the prediction of liver disease.

Conclusions

In summary, this study has developed and externally validated the ALFI model for prediction of liver disease diagnosis in patients with no clinically obvious liver disease having their LFTs taken within primary care. From this model, a simple scoring tool was developed to facilitate GP decision making with regards to retesting or referring their patients. Its main

For peer review only

strength is to accurately 'rule out' patients who have low risk of being diagnosed with a liver disease. This has the potential to save health service costs and prevent unnecessary further investigations and treatments.

Acknowledgements

The authors wish to thank Alison Bell from HIC for extracting and pseudonymising all the datasets used. We thank Primary Care Clinical Informatics Unit (PCCIU) at the University of Aberdeen for providing the Practice Team Initiative cohort for external validation of our model.

Funding

This work was supported by the UK National Health Service Research & Development Programme Health Technology Assessment Programme (project number 03/38/02) and supported by the Backett Weir Russell Career Development Fellowship.

Department of Health Disclaimer: The views and opinions expressed therein are those of the authors and do not necessarily reflect those of the Department of Health.

The study funder did not participate in the study design; in the collection, analysis and interpretation of data; in the writing of the report; or in the decision to submit the article for publication.

Competing interests

None declared.

Authors' contributions

For peer review only

DJM contributed to the design of the study, conducted the analyses, drafted and finished the paper. JFD, FMS, PR, WR and SR contributed to the design and conduct of the study, and the writing of the paper. PTD contributed to, and oversaw the design and conduct of the study, and contributed to the writing of the paper. He is the guarantor for the paper. All authors read and approved the final manuscript.

References

1. Pratt DS, Kaplan MM: **Evaluation of abnormal liver-enzyme results in asymptomatic patients**. *N Engl J Med* 2000, **342**:1266-1271.

2. Schalk BWM, Visser M, Bremmer MA, Penninx BWJH, Bouter LM, Deeg DJH: **Change of serum albumin and risk of cardiovascular disease and all-cause mortality: Longitudinal Aging Study Amsterdam**. *Am J Epidemiol* 2006, **164**:969-977.

3. Sorensen HT, Moller-Petersen JF, Felding P, Andreasen C, Nielsen JO: **Epidemiology of abnormal liver function tests in general practice in a defined population in Denmark**. *Dan Med Bull* 1991, **38**:420-422.

4. Kim HC, Nam CM, Jee SH, Han KH, Oh DK, Suh I: **Normal serum aminotransferase concentration and risk of mortality from liver diseases: Prospective cohort study**. *Br Med J* 2004, **328**:983-986.

5. Roderick P: **Commentary: Liver function tests: defining what's normal**. *Br Med J* 2004, **328**:987.

6. Sherwood P, Lyburn I, Brown S, Ryder S: **How are abnormal results for liver function tests dealt with in primary care? Audit of yield and impact**. *Br Med J* 2001, **322**:276-278.

7. McLernon DJ, Donnan PT, Ryder S, Roderick P, Sullivan FM, Rosenberg W, Dillon JF: **Health outcomes following liver function testing in primary care: a retrospective cohort study**. *Fam Pract* 2009, **26**:251-259.

For peer review only

8. Moons K, Royston P, Vergouwe Y, Grobbee D, Altman D: **Prognosis and prognostic research: what, why, and how?** *Br Med J* 2009, **338**:b375.

9. Fornasini M, Brotons C, Sellarès J, Martinez M, Galán ML, Sáenz L, da Pena JM: **Consequences of using different methods to assess cardiovascular risk in primary care**. *Fam Pract* 2006, **23**:28-33.

10. Siris ES, Baim S, Nattiv A: **Primary care use of FRAX: absolute fracture risk assessment in postmenopausal women and older men**. *Postgrad Med* 2010, **122**:82-90.

11. Altman DG, Vergouwe Y, Royston P, Moons KGM: **Prognosis and prognostic research: validating a prognostic model**. *Br Med J* 2009, **338**:b605.

12. Donnan PT, McLernon D, Dillon JF, Ryder S, Roderick P, Sullivan F, Rosenberg W: **Development of a decision support tool for primary care management of patients with abnormal liver function tests without clinically apparent liver disease: a record-linkage population cohort study and decision analysis (ALFIE)**. *Health Technol Assess* 2009, **13**(25).

13. Carstairs V, Morris R: **Deprivation and mortality: an alternative to social class?** *Community Med* 1989, **11**:210-219.

14. Evans JMM, MacDonald TM: **Record-linkage for pharmacovigilance in Scotland**. *Br J Clin Pharm* 1999, **47**:105-110.

15. NHS National Services Scotland: **SMR01 – General/Acute Inpatient and Day Case** [http://www.datadictionary.scot.nhs.uk/isddd/9065.html]

16. Morris AD, Boyle DI, MacAlpine R, Emslie-Smith A, Jung RT, Newton RW, MacDonald TM: **The diabetes audit and research in Tayside Scotland (darts) study: electronic record linkage to create a diabetes register**. *Br Med J* 1997, **15**:524-528.

17. Donnan PT, Wei L, Steinke DT, Phillips G, Clark R, Noone A, Sullivan FM, Macdonald TM, Davey PG: **Presence of bacteriuria caused by trimethoprium resistant bacteria in**

For peer review only

patients prescribed antibiotics: multilevel model with practice and individual patient data. *Br Med J* 2004, **328**:1297-1301.

18. Steinke DT, Weston TL, Morris AD, MacDonald TM, Dillon JF: **The epidemiology of liver disease in Tayside database: a population-based record-linkage study**. *J Biomed Inform* 2003, **35**:186-193.

19. Rubin DB: *Multiple imputation for nonresponse in surveys*. New York: Wiley; 1987.

20. Chambless LE, Cummiskey CP, Cui G: **Several methods to assess improvement in risk prediction models: extension to survival analysis**. *Stat Med* 2011, **30**:22-38.

21. Pencina MJ, D'Agostino RB: **Overall C as a measure of discrimination in survival analysis: model specific population value and confidence interval estimation**. *Stat Med* 2004, **23**:2109-2123.

22. Steyerberg EW: *Clinical prediction models: a practical approach to development, validation, and updating*. New York: Springer; 2009.

23. University of Aberdeen: **Primary Care Clinical Informatics Unit** [http://www.abdn.ac.uk/pcciu/index.htm]

24. Information Services Division Scotland: **General Practice – Practice Team Information (PTI)** [http://www.isdscotland.org/isd/1283.html#Background_to_PTI]

25. Sullivan LM, Massaro JM, D’Agostino Sr RB: **Tutorial in biostatistics: Presentation of multivariate data for clinical use: The Framingham Study risk score functions**. *Stat Med* 2004, **23**:1631-1660.

26. McLernon DJ, Dillon JF, Sullivan FM, Roderick P, Rosenberg WM, Ryder S, Donnan PT: **The utility of liver function tests for mortality prediction within one year in primary care using the Algorithm for Liver Function Investigations (ALFI)**. *PLoS ONE* 2012, **7**:e50965.

27. Dorans K: **Pilot projects aim to ease access to clinical data**. *Nature Med* 2009, **15**: 226.

For peer review only

28. D'Agostino RB, Grundy S, Sullivan LM, Wilson P: **Validation of the Framingham Coronary Heart Disease Prediction Scores. Results of a multiple ethic groups investigation**. *JAMA* 2001, **286**:180-187.

29. Hawkins RC. **The evidence based medicine approach to diagnostic testing: practicalities and limitations**. *Clin Biochem Rev* 2005, **26**:7-18.

30. Craig P, Dieppe P, Macintyre S, Mitchie S, Nazareth I, Petticrew M. **Developing and evaluating complex interventions: the new Medical Research Council guidance**. *Br Med J* 2008, **337**:a1655.

For peer review only

Box 1: Databases record-linked to create the Tayside derivation cohort

1. Death registry from the General Register Office for Scotland including date and causes of death.
2. Deprivation score was assessed by the Carstairs method [13]. The score was divided into two categories of affluent and deprived.
3. Tayside Community Health Index (CHI) files. Contains CHI number [14], name, address and date of birth for all individuals registered with a general practitioner in Tayside. It also holds migration dates to and from Tayside. The database was pseudonymised before release to the researcher.
4. Regional biochemistry database. All LFTs came from the largest hospital laboratory in the Tayside region, Ninewells. Two smaller hospital laboratories contributed electronic LFT results later, one from 1998 and the other from 2003.
5. Scottish Morbidity Records 1 (SMR01) database: Patient specific morbidity data are routinely collected in Scotland and collectively these are known as Scottish Morbidity Records (SMR). SMR01 contains hospital admissions and procedures for all hospitals in Scotland and is one of the oldest and most complete national health datasets in the world [15]. Major co-morbidity groups at baseline were identified using this database, and the Diabetes Audit and Research Tayside Scotland (DARTS) and Heart disease Evidence-based Audit and Research Tayside Scotland (HEARTS) databases for diabetes and chronic heart disease respectively [16].
6. SMR04 database: Holds all inpatient and day case episodes for mental health specialties, which identified patients admitted/discharged with diagnoses of alcohol dependency or drug misuse.
7. Pharmacist dispensed prescription database: A community based database that holds encashed prescriptions in Tayside [17], and allowed identification of patients on some potentially hepatotoxic drugs including statins, antibiotics, and Non-Steroidal Anti-Infammatory Drugs (NSAIDs).
8. Epidemiology of Liver Disease In Tayside (ELDIT) database: Contains all Tayside patients with liver diseases that have been 'electronically' diagnosed using record-linkage of biomedical datasets, including virology and immunology [18]. ELDIT was used for the study exclusion criteria (i.e. previous liver disease, liver disease complications within 6 weeks) and for the outcome of liver disease diagnosis during the two-year follow-up period.

Table 1 Baseline characteristics of patients in the derivation (n=95977) and validation (n=11653) cohorts

Baseline Characteristics		Cohort n(%) *or* Median (IQR)	
		Derivation	Validation
Age (years)		54.6 (39.2-68.8)	60.0 (47.0, 72.0)
Gender	Male	40374 (42.1)	5271 (45.2)
	Female	55603 (57.9)	6382 (54.8)
Carstairs category[1]	Affluent	47286 (49.3)	2753 (23.6)
	Deprived	48691 (50.7)	8900 (76.4)
Comorbidity history	Cancer[2]	3629 (3.8)	956 (8.2)
	Diabetes	1386 (1.4)	1441 (12.4)
	IHD	5370 (5.6)	2034 (17.5)
	Renal disease	141 (0.2)	155 (1.3)
	Respiratory disease	2636 (2.8)	883 (7.6)
	Stroke	1471 (1.5)	583 (5.0)
Medication in previous 3 months	Statins	3176 (3.3)	3178 (27.3)
	NSAIDs	6698 (7.0)	1762 (15.1)
	Antibiotics	8307 (8.7)	1962 (16.8)
Abusive substance	Alcohol	2632 (2.7)	465 (4.0)
	Drug	371 (0.4)	0 (0.0)
	Methadone	377 (0.4)	10 (0.1)
Liver function tests	Albumin (g/L)	44.0 (42.0-46.0)	44.0 (41.0, 46.0)
	ALP (U/L)	76.0 (62.0-94.0)	75.0 (62.0, 92.0)
	Transaminase (U/L)	18.0 (14.0-26.0)	21.0 (16.0, 30.0)
	GGT (U/L)	26.0 (17.0-47.0)	27.0 (18.0, 45.0)
	Bilirubin[3]		
	Normal	81111 (91.0)	10587 (90.8)
	Mildly raised	8058 (9.0)	1066 (9.2)

Data reported are median (interquartile range) or percentage

For peer review only

[1]Carstairs categories 1 to 3 were recoded as affluent and categories 4 to 7 were recoded as deprived.

[2]Not including biliary cancer or hepatocellular cancer;

[3]Normal bilirubin defined as 0-15□mol/L for females, 0-17µmol/L for males; Mildly raised bilirubin defined as 16-35 µ mol/L for females, 18-35 µ mol/L for males.

IQR interquartile range; IHD ischaemic heart disease; NSAID non-steroidal anti-inflammatory; ALP alkaline phosphatase; GGT gamma-glutamyltransferase.

For peer review only

Table 2 Parameter estimates (95% CI) and IDI for the final log-normal regression model predicting risk of a liver disease diagnosis within 2 years of initial liver function tests (481 diagnosed)

Parameter	Coefficient (95% CI)	P-value	IDI, %
Intercept	9.524 (1.986 to 17.062)	0.013	
Albumin	0.488 (0.306 to 0.669)	<0.001	0.711[a]
Log(GGT)	-1.704 (-2.223 to -1.184)	<0.001	0.689
Methadone (yes *v* no)	8.319 (-2.019 to 18.657)	0.115	0.465[a]
Log(ALP)	-0.739 (-1.213 to -0.264)	0.002	0.307
Log(transaminase)	2.016 (-0.151 to 4.183)	0.068	0.266[a]
Alcohol dependent (yes *v* no)	-1.210 (-1.856 to -0.563)	<0.001	0.143
Gender (Male *v* Female)	0.583 (0.236 to 0.930)	0.001	0.135
Age at baseline	0.014 (0.004 to 0.024)	0.009	0.121
Deprived[b] (yes *v* no)	-0.518 (-0.852 to -0.183)	0.002	0.013
Methadone X Albumin	-0.295 (-0.530 to -0.061)	0.014	
Albumin X Log(transaminase)	-0.070 (-0.122 to -0.018)	0.008	
Scale	4.551 (4.192 to 4.910)	<0.001	

GGT = gamma-glutamyltransferase; ALP = alkaline phosphatase; IDI=integrated discrimination improvement.

[a] the relative interaction terms containing this parameter were also excluded.

[b] Carstairs categories 1 to 3 were coded as affluent and categories 4 to 7 were coded as deprived.

Note: Parameter estimates are in decreasing order of IDI. A negative coefficient indicates an increased risk of diagnosis, whilst a positive coefficient indicates a decreased risk of diagnosis. However, this differs for terms involved in interactions i.e. increasing transaminase increases risk; for a methadone user, increasing albumin increases risk; for non-methadone users, decreasing albumin increases risk.

For peer review only

Figure titles and legends

Figure 1. Probability of a liver disease diagnosis during the two years for the average risk patient and two example patients with different risk levels.

Note: Average risk is defined as the risk calculated using the average linear predictor value from the model. Patient 1 and Patient 2 characteristics are presented in the boxes within the plot. The estimated risk of being diagnosed with a liver disease within two years for each of these patients is 0.002 (average risk patient), 0.097 (Patient 1) and 0.493 (Patient 2).

Figure 2. Clinical scoring tool for likely liver disease diagnosis in primary care.

For each risk factor category enter the corresponding score into the box on the right hand side. Sum the scores in the total score box. Look for the total score in the lower table and read off the risk of liver disease within 6 months and/or 2 years.

For peer review only

List of Supplementary Materials

File name: Appendix 1

File format: .docx

Title of data: Liver diseases from the ELDIT database

Description of data: Liver diseases from the ELDIT database used as the outcome in the model and comorbidities used as potential predictors of the outcome along with their original clinical database source(s)

File name: Appendix 2

File format: .docx

Title of data: Calibration plots

Description of data: Calibration plots for the final model applied to the development cohort and to the validation cohort

File name: Appendix 3

File format: .docx

Title of data: Performance of the final model to predict liver disease diagnosis

Description of data: Receiver operating characteristic (ROC) curves for the final model to predict liver disease diagnosis applied to the derivation cohort and the external cohort. Sensitivity, specificity, positive predictive value (PPV), and negative predictive value (NPV) for different percentiles of liver disease risk for the derivation and validation cohorts are also presented.

File name: Appendix 4

File format: .docx

For peer review only

Title of data: Calculating the risk of liver disease diagnosis within two years from the final model.

Description of data: A mathematical formula showing how to calculate the predicted risk of liver disease diagnosis at any time point during a two year maximum follow-up period. A worked example is also presented.

For peer review only

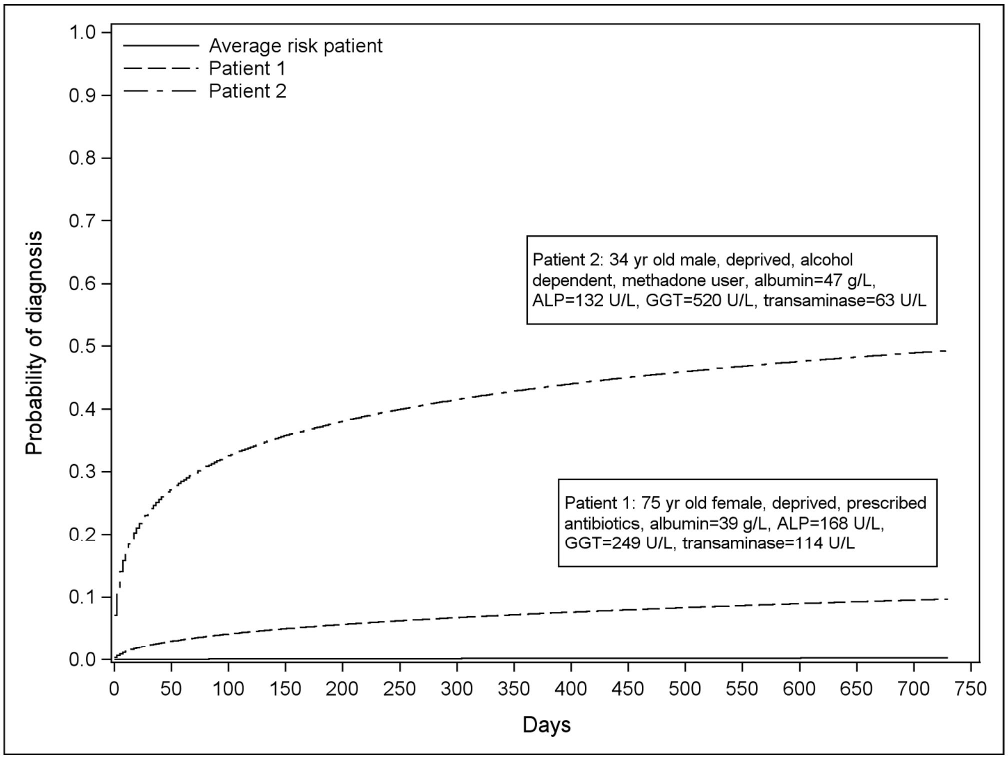

508x381mm (300 x 300 DPI)

Risk factors	Categories and corresponding score						Score
Age (Years)	16 to 35	36 to 55	56 to 75	≥75			
	+1	0	-1	-2			
Transaminase (U/L)	<20	20 to 39	40 to 59	60 to 79	≥80		
	0	+3	+5	+6	+7		
ALP (U/L)	<60	60 to 89	90 to 119	120 to 149	150 to 179	≥180	
	0	+1	+2	+3	+3	+4	
GGT (U/L)	<30	30 to 59	60 to 89	90 to 119	120 to 149	≥150	
	0	+6	+9	+12	+13	+14	
Albumin (g/L) AND	<31	31 to 35	36 to 40	41 to 45	≥46		
Methadone user	+19	+20	+21	+21	+22		
OR							Or
Not methadone user	+19	+15	+10	+5	0		
Gender	Male	Female					
	-2	0					
Deprived	No	Yes					
	0	+2					
Alcohol dependent	No	Yes					
	0	+5					
						Total score	

Estimated probability of liver disease diagnosis at 6 months and 2 years

Total Score	Liver disease probability (%)		Total Score	Liver disease probability (%)	
	within 6 months	within 2 years		within 6 months	within 2 years
5 or less	<0.1	<0.1	26	1.3	2.7
6	<0.1	0.1	27	1.5	3.1
7	<0.1	0.1	28	1.8	3.6
8	0.1	0.1	29	2.0	4.1
9	0.1	0.2	30	2.3	4.6
10	0.1	0.2	31	2.7	5.2
11	0.1	0.3	32	3.1	5.9
12	0.1	0.3	33	3.5	6.6
13	0.1	0.4	34	4.0	7.4
14	0.2	0.4	35	4.5	8.2
15	0.2	0.5	36	5.1	9.2
16	0.2	0.6	37	5.7	10.2
17	0.3	0.7	38	6.4	11.2
18	0.4	0.8	39	7.2	12.4
19	0.4	1.0	40	8.0	13.6
20	0.5	1.2	41	9.0	15.0
21	0.6	1.3	42	10.0	16.4
22	0.7	1.6	43	11.0	17.9
23	0.8	1.8	44	12.2	19.5
24	1.0	2.1	45 or more	>13.3	>21.0
25	1.1	2.4			

203x259mm (300 x 300 DPI)

Appendix 1 Liver diseases from the ELDIT database used as the outcome and comorbidities used as potential predictors of the outcome along with their original clinical database source(s)

Disease	Hospital admission diagnosis codes from SMR01 database		Other source databases from Ninewell's hospital
	ICD 9	ICD 10	
**Liver disease diagnosis**			
Hepatitis B			Virology
Hepatitis C			Virology
Autoimmune hepatitis	571.4	K73.0, K73.2, K73.8, K73.9	+ve Pathology, immunology (+ve ASM)
Cirrhosis	571.2, 571.5, 571.6	K70.3, K74.3-K74.6, K76.1	Pathology
Primary biliary cirrhosis:			
Definite			+ve Pathology, immunology (+ve AMA), biochemistry (↑GGT, ↑Alk Phos)
Probable			Any two positive tests from the above three.
Possible			Immunology (+ve AMA)
Alcoholic cirrhosis	As cirrhosis + 291, 303, 305.0	As cirrhosis + F10	SMR1/SMR4 ICD codes for alcohol
Alcoholic hepatitis	571.1	K70.1	Pathology
Alcoholic related liver disease	Any liver disease codes+alcohol codes	Any liver disease codes+alcohol codes	Pathology
Fatty liver disease	571.0, 571.8	K70.0, K76.0	Pathology
Hepatocellular carcinoma	155.0, 155.2	C22.0, C22.2-C22.9	Pathology (ICD from SMR1/SMR6)
Wilson's disease	275.1	E83.0	
Haemochromatosis	275.0, 285.0	D64.2, E83.1	Pathology
Alpha 1-antitrypsin			Biochemistry (+ve Alpha 1-antitrypsin)
**Complications of liver disease**			
Oesophageal Varices	456.1, 456.2	I85.9, I98.2A	Endoscopy records
- bleeding	456.0	I85.0	
Gastric varices		I86.4	
Ascites	789.5	R18X	
Encephalopathy	572.2	K72.9	
Portal hypertension	Any complication above or 572.3	Any complication or K76.6	
**Other liver conditions**			
Acute/subacute necrosis	570.9		
Alcoholic liver damage, unspecified	571.3	K70.9	
Chronic hepatitis	571.4		
Non-alcoholic chronic liver disease, unspecified	571.9		
Abscess of liver	572.0	K75.0	

For peer review only

Hepatorenal syndrome	572.4	K76.7	
Other sequelae of chronic liver disease	572.8		
Other liver disorder eg hepatoptosis	573.8	K76.8	
Unspec liver disorder	573.9	K76.9	
Alcoholic hepatic failure		K70.4	
Acute/subacute hepatic failure		K72.0	
Chronic hepatic failure		K72.1	
Occlusion of vena cava	453.2	I82.2	
Portal vein thrombosis	452	I81	
Hepatitis B	070	B16, B18.0, B18.1	
Hepatitis C	070	B17.1, B18.2	
Other viral hepatitis	070	B17-19	
**Comorbidities**			
Ischaemic heart disease	410-414	I20-I25	
Other cancers	140-208, 230-234 (exclude cholangiocarcinoma and hepatocellular codes)	C00-D09 (exclude cholangiocarcinoma and hepatocellular codes)	
Diabetes	250	E10-E14	
Respiratory	466, 480-496	J10-J18, J20, J40X-J47X, J66-J67	
Renal	584-586	N17-N19X, I12.0	
Stroke	430-438	I60-	

ICD=International Classification of Diseases; ASM=anti-smooth muscle antibody; AMA=antimitochondrial antibody; GGT; Alk phos=alkaline phosphatase;

Note: All diseases are definite diagnoses apart from Primary Biliary Cirrhosis which was coded as either definite, probable or possible.

For peer review only

Appendix 2: Calibration plots

In order to draw a calibration plot we have to first of all perform some calculations. First of all divide the cohort into intervals (each with 100 patients) of increasing risk of liver disease and calculating the mean *predicted* risk of liver disease at two years for each interval. Secondly, calculate the mean *actual* risk of liver disease using the Kaplan-Meier method for each interval. The mean predicted risks were then plotted against the mean actual risks. A model with perfect calibration would see the points lie along the diagonal.

Figure A1 shows reasonable agreement between the predicted and actual risks for the model in the derivation cohort.

Figure A1: Calibration plot showing the relationship between the predicted and actual (Kaplan-Meier) risks of liver disease diagnosis for the final model applied to the derivation cohort

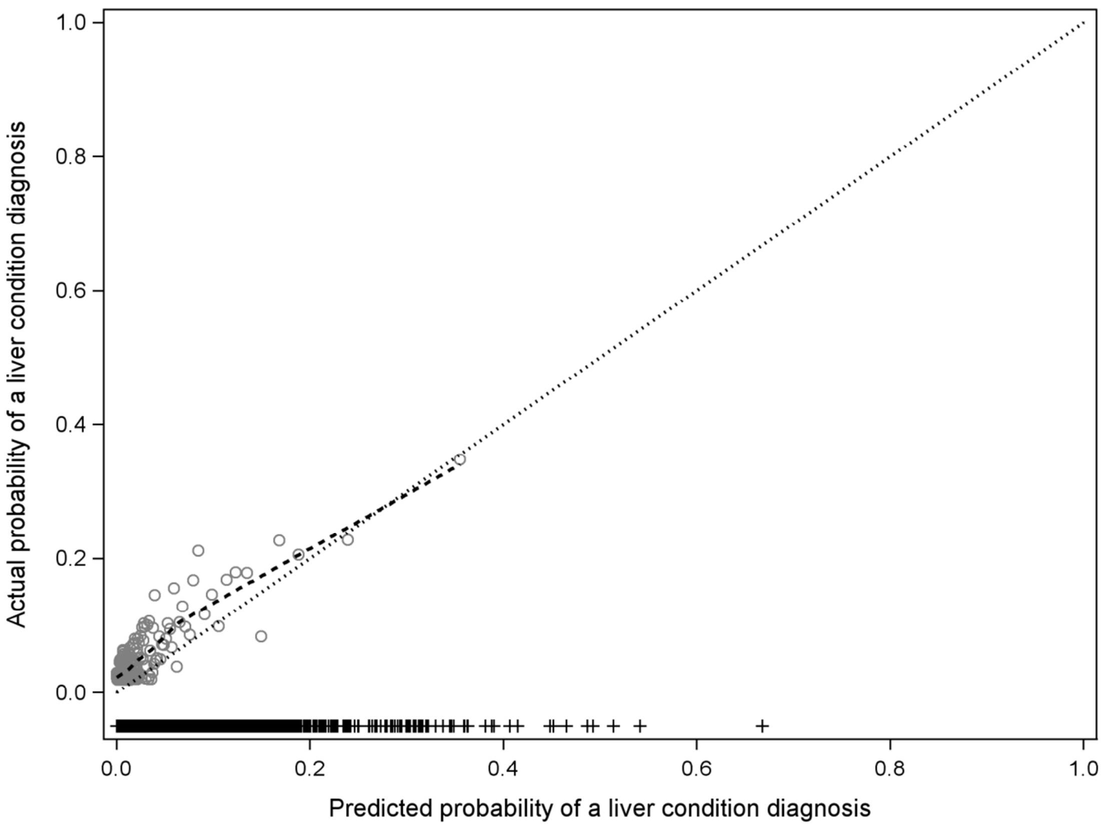

Note: Patients were ranked into order of predicted risk within two years and divided into groups of 100 patients. The circles represent the mean risks for each group of 100 patients; the dotted line represents the perfect relationship; the dashed line represents the smooth non-parametric Loess calibration curve fitted through the circles; the plus symbols represent the spread of patients across predicted risks.

The calibration plot for the model applied to the external validation cohort (Figure A2) showed some visible evidence of over-fitting for the highest risk group, however the calibration slope showed no significant deviation from 1 (slope = 0.871 (95% CI 0.696 to 1.047); p=0.15).

Figure A2: Calibration plot showing the relationship between the predicted and actual risks of survival for the final model applied to the external validation cohort

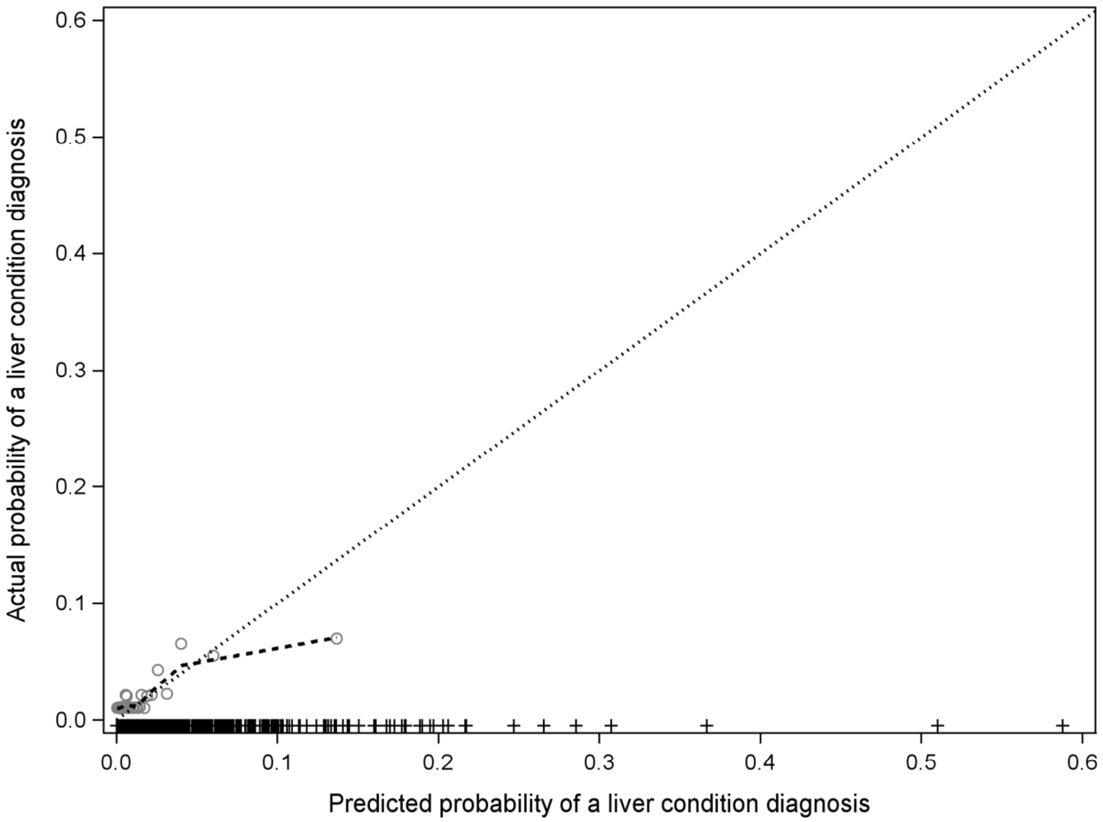

Note: Dashed line represents the smooth non-parametric Loess calibration curve; the circles represent the mean risks for subgroups of patients; the dotted line represents the perfect relationship; the plus symbols represent the spread of patients across predicted risks.

Appendix 3 Performance of the final model to predict liver disease diagnosis

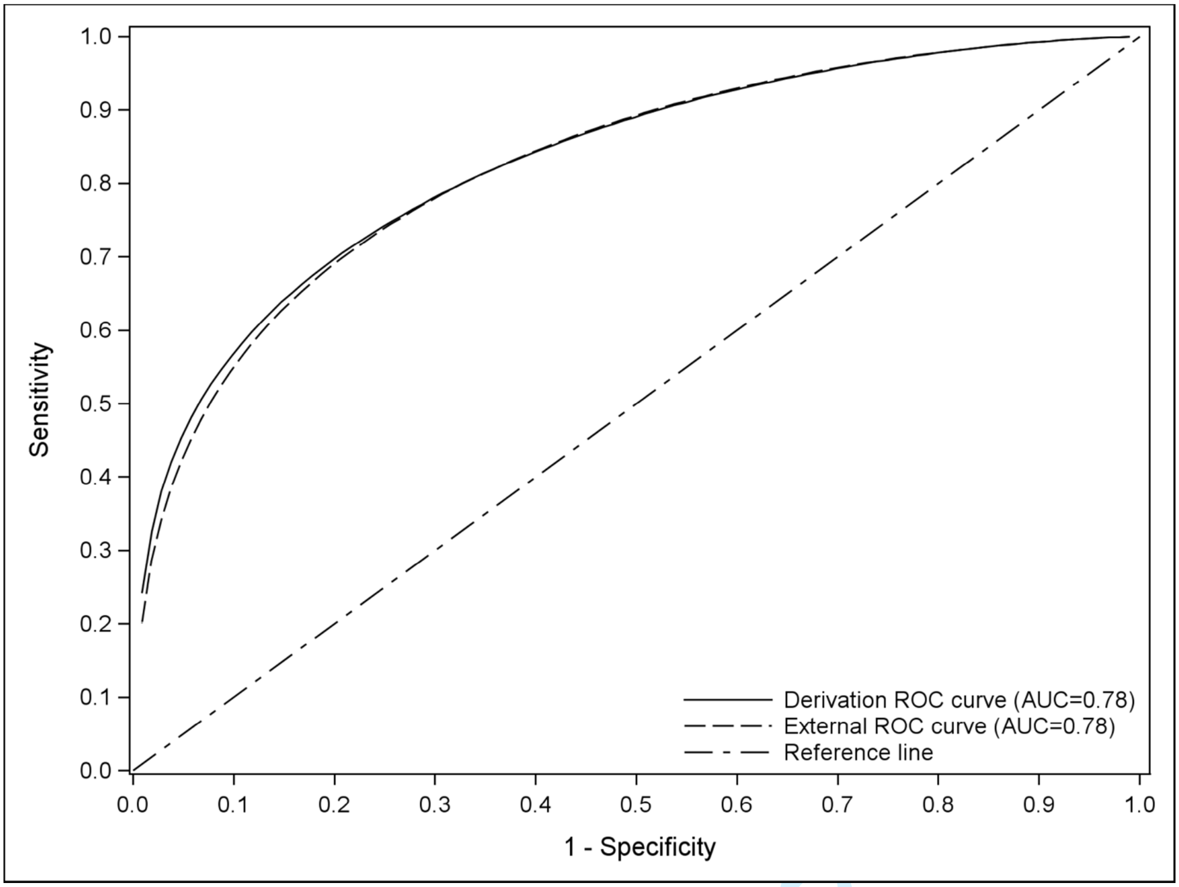

Figure A3 Receiver operating characteristic (ROC) curves for the final model to predict liver disease diagnosis in the derivation cohort and external cohort.

only

Table A1 Final model sensitivity, specificity, positive predictive value (PPV), and negative predictive value (NPV) for different percentiles of liver disease risk for the derivation and validation cohorts.

Derivation cohort						Validation cohort					
Cut-off for predicted risk,%	Number (%) of patients over cut-off (out of 95,977)	Sens, %	Spec, %	PPV, %	NPV, %	Cut-off for predicted risk,%	Number (%) of patients over cut-off (out of 11653)	Sens, %	Spec, %	PPV, %	NPV, %
≥0.215[a]	47989 (50)	89.0	50.2	0.903	99.8	≥0.269[a]	5827 (50)	89.2	50.2	1.14	99.9
≥0.427[b]	23995 (25)	74.1	75.2	1.50	99.8	≥0.569[b]	2914 (25)	73.7	75.3	1.88	99.8
≥0.881[c]	9598 (10)	56.5	90.2	2.87	99.8	≥1.22[c]	1167 (10)	54.5	90.3	3.46	99.7
≥1.52[d]	4799 (5)	45.3	95.2	4.59	99.7	≥2.08[d]	583 (5)	42.1	95.2	5.35	99.6
≥5	1155 (1.20)	26.3	98.9	11.1	99.6	≥5	185 (1.59)	31.2	99.4	8.14	99.5
≥5.82[e]	960 (1.00)	24.2	99.1	12.2	99.6	≥6.95[e]	117 (1.00)	20.1	99.1	12.6	99.5
≥10	463 (0.48)	16.5	99.6	17.3	99.6	≥10	54 (0.46)	19.2	100.0	13.1	99.4
≥20	110 (0.11)	6.53	99.9	28.7	99.5	≥20	12 (0.10)	8.03	100.0	23.8	99.4
≥30	38 (0.04)	2.93	100	36.5	99.5	≥30	5 (0.04)	4.54	100.0	30.6	99.4
≥40	10 (0.01)	1.00	100	44.4	99.5	≥40	2 (0.02)	2.47	100.0	36.6	99.4

Cut-offs for: [a]50th; [b]75th; [c]90th; [d]95th; [e]99th percentile.

For peer review only

Appendix 4: Calculating the risk of liver disease diagnosis within two years from the final model.

**Predictor**	**Description**	**Possible values**
Gender	Male or female	=1 if Male<br>=0 if Female
Age	Age	16 to 103
Deprived	Deprivation based on Carstairs category linked to postcode	=1 if Yes (Carstairs categories 4 to 7)<br>=0 if No (Carstairs categories 1 to 3)
Alcohol	Previous hospital admission for alcohol dependency	=1 if Yes<br>=0 if No
Methadone	Ever prescribed methadone	=1 if Yes<br>=0 if No
ALP	Alkaline phosphatase result	9U/L to ∞
GGT	Gamma-glutamyltransferase result	2U/L to ∞
Albumin	Albumin result	14 to 63g/L
Transaminase	Alanine transaminase/aspartate aminotransferase result	4U/L to ∞

First calculate the linear predictor, ***Xβ***, using the possible values contained within the table above:
Xβ=9.5239 – ***A*** + ***B***

where:

A= 0.5179*Deprived + 1.2097*Alcohol + 0.7385*LOG_e(ALP) + 1.7036*LOG_e(GGT+0.5) + 0.0700*Albumin*LOG_e(Transaminase) + 0.2954*Methadone*Albumin

and

B= 0.5827*Gender + 0.0138*Age + 8.3192*Methadone + 2.0162*LOG_e(Transaminase) + 0.4876*Albumin

Now, calculate Z:

$$Z = \frac{LOG_e(T) - X\beta}{4.5509}$$

Where ***T***= follow-up time, choose from 1 to 730 days

Predicted risk of liver disease within *T* days = Φ(***Z***),
i.e. the probability of Z from the standard Normal distribution. This can be found using a software package such as Microsoft Excel. In Excel use the following function: NORMSDIST(***Z***).

For peer review only

A worked example

Suppose we want to calculate the risk of liver disease within 2 years for a 34 year-old male methadone user living in a deprived area. He has the following LFT results: ALP=132U/L, albumin=40g/L, GGT=150U/L and Transaminase=63U/L

Xβ=9.5239 – ***A*** + ***B***

where:

A= 0.5179*1 + 1.2097*0 + 0.7385*LOG_e(132) + 1.7036*LOG_e(150.5) + 0.0700*40*LOG_e(63) + 0.2954*1*40

and

B= 0.5827*1 + 0.0138*34 + 8.3192*1 + 2.0162*LOG_e(63) + 0.4876*40

Xβ=9.5239 – 36.0824 + 37.2285

=10.67

$$Z = \frac{\mathrm{LOG}(730) - 10.67}{4.5509}$$

= -0.8959

Predicted risk of liver disease within 730 days = Φ(**-0.8959**) = **0.1852** using the function NORMSDIST(Z) in Excel (i.e. NORMSDIST(-0.8959)).

For peer review only

TITLE: Prediction of liver disease diagnosis in primary care using the Algorithm for Liver Function Investigations (ALFI): model development and validation
Supplementary file

STROBE Statement—Checklist of items that should be included in reports of ***cohort studies***

	Item No	Recommendation	Location within article
**Title and abstract**	1	(*a*) Indicate the study's design with a commonly used term in the title or the abstract	Page 1 (title) Page 2 (abstract)
		(*b*) Provide in the abstract an informative and balanced summary of what was done and what was found	Page 2
**Introduction**			
Background/rationale	2	Explain the scientific background and rationale for the investigation being reported	Page 4
Objectives	3	State specific objectives, including any prespecified hypotheses	Page 4-5
**Methods**			
Study design	4	Present key elements of study design early in the paper	Pages 7 and 8
Setting	5	Describe the setting, locations, and relevant dates, including periods of recruitment, exposure, follow-up, and data collection	Pages 7 and 8 (Derivation cohort)
Participants	6	(*a*) Give the eligibility criteria, and the sources and methods of selection of participants. Describe methods of follow-up	Pages 5 and 6
		(*b*) For matched studies, give matching criteria and number of exposed and unexposed	n/a
Variables	7	Clearly define all outcomes, exposures, predictors, potential confounders, and effect modifiers. Give diagnostic criteria, if applicable	Page 7 (Baseline characteristics & Outcomes)
Data sources/ measurement	8*	For each variable of interest, give sources of data and details of methods of assessment (measurement). Describe comparability of assessment methods if there is more than one group	Pages 6 and 21 (Databases and Box 1)
Bias	9	Describe any efforts to address potential sources of bias	Pages 8 and 9 (Model derivation and validation)
Study size	10	Explain how the study size was arrived at	n/a (whole population)
Quantitative variables	11	Explain how quantitative variables were handled in the analyses. If applicable, describe which groupings were chosen and why	Pages 7 and 8 (Baseline characteristics, Outcomes, Model derivation)
Statistical methods	12	(*a*) Describe all statistical methods, including those used to control for confounding	Pages 7-9 Further detail in web appendix (methods section p1-3)
		(*b*) Describe any methods used to examine subgroups and interactions	Page 8 (Model derivation)
		(*c*) Explain how missing data were addressed	Page 8 (Model derivation)

For peer review only - http://bmjopen.bmj.com/site/about/guidelines.xhtml

		(*d*) If applicable, explain how loss to follow-up was addressed	Page 7-8 (Model derivation)
		(*e*) Describe any sensitivity analyses	Page 9 (Model validation)
**Results**			
Participants	13*	(a) Report numbers of individuals at each stage of study—eg numbers potentially eligible, examined for eligibility, confirmed eligible, included in the study, completing follow-up, and analysed	Page 10
		(b) Give reasons for non-participation at each stage	As above
		(c) Consider use of a flow diagram	n/a
Descriptive data	14*	(a) Give characteristics of study participants (eg demographic, clinical, social) and information on exposures and potential confounders	Pages 10 and 22 (Baseline characteristics and Table 1)
		(b) Indicate number of participants with missing data for each variable of interest	Page 10
		(c) Summarise follow-up time (eg, average and total amount)	Page 10 (Liver disease diagnosis)
Outcome data	15*	Report numbers of outcome events or summary measures over time	Page 10 (Liver disease diagnosis)
Main results	16	(*a*) Give unadjusted estimates and, if applicable, confounder-adjusted estimates and their precision (eg, 95% confidence interval). Make clear which confounders were adjusted for and why they were included	Pages 11-12, 24 (only adjusted models presented)
		(*b*) Report category boundaries when continuous variables were categorized	Page 10 (Baseline characteristics) & Page 23 and 24 (footnotes to Table 1 and 2)
		(*c*) If relevant, consider translating estimates of relative risk into absolute risk for a meaningful time period	n/a
Other analyses	17	Report other analyses done—eg analyses of subgroups and interactions, and sensitivity analyses	Page 12 (validation) & web appendix (Appendices 2 and 3)
**Discussion**			
Key results	18	Summarise key results with reference to study objectives	Page 13
Limitations	19	Discuss limitations of the study, taking into account sources of potential bias or imprecision. Discuss both direction and magnitude of any potential bias	Pages 13-14
Interpretation	20	Give a cautious overall interpretation of results considering objectives, limitations, multiplicity of analyses, results from similar studies, and other relevant evidence	Pages 14-15
Generalisability	21	Discuss the generalisability (external validity) of the study results	Pages 14-15 (External validation text) & web appendices 2 and 3.

For peer review only

For peer review only - http://bmjopen.bmj.com/site/about/guidelines.xhtml

**Other information**			
Funding	22	Give the source of funding and the role of the funders for the present study and, if applicable, for the original study on which the present article is based	Page 16

*Give information separately for exposed and unexposed groups.

Note: An Explanation and Elaboration article discusses each checklist item and gives methodological background and published examples of transparent reporting. The STROBE checklist is best used in conjunction with this article (freely available on the Web sites of PLoS Medicine at http://www.plosmedicine.org/, Annals of Internal Medicine at http://www.annals.org/, and Epidemiology at http://www.epidem.com/). Information on the STROBE Initiative is available at http://www.strobe-statement.org.

For peer review only

Prediction of liver disease in patients whose liver function tests have been checked in primary care: model development and validation using population-based observational cohorts

Journal:	*BMJ Open*
Manuscript ID:	bmjopen-2014-004837.R1
Article Type:	Research
Date Submitted by the Author:	02-May-2014

Complete List of Authors:	McLernon, David; University of Aberdeen, Division of Applied Health Sciences<br>Donnan, Peter; University of Dundee, Dundee Epidemiology and Biostatistics Unit<br>Sullivan, Frank; University of Toronto, Department of Community and Family Medicine<br>Roderick, Paul; University of Southampton, Faculty of Medicine<br>Rosenberg, William; University College London, Centre for Hepatology, Division of Medicine and ULCH-UCL NIHR Biomedical Research Centre,<br>Ryder, Stephen; Nottingham University Hospitals NHS Trust and Biomedical Research Unit, Department of Gastroenterology<br>Dillon, John; University of Dundee, Ninewells Hospital and Medical School

**Primary Subject Heading**:	General practice / Family practice
Secondary Subject Heading:	Gastroenterology and hepatology, Epidemiology
Keywords:	PRIMARY CARE, Hepatology < INTERNAL MEDICINE, EPIDEMIOLOGY

SCHOLARONE™ Manuscripts

Prediction of liver disease in patients whose liver function tests have been checked in primary care: model development and validation using population-based observational cohorts

David J McLernon[1*]
* Corresponding author
Email: d.mclernon@abdn.ac.uk

Peter T Donnan[2]
Email: p.t.donnan@dundee.ac.uk

Frank M Sullivan[3]
Email: frank.sullivan@nygh.on.ca

Paul Roderick[4]
Email: pjr@soton.ac.uk

William M Rosenberg[5]
Email: w.rosenberg@ucl.ac.uk

Steve D Ryder[6]
Email: Stephen.Ryder@nuh.nhs.uk

John F Dillon[7]
Email: j.dillon@nhs.net

[1]Medical Statistics Team, Division of Applied Health Sciences, College of Life Sciences and Medicine, University of Aberdeen, UK

[2]Dundee Epidemiology and Biostatistics Unit, Division of Population Health Sciences, Medical Research Institute, University of Dundee, Dundee, UK

[3]Department of Community and Family Medicine, University of Toronto, Canada
[4]Academic Unit of Primary Care and Population Sciences, Faculty of Medicine, University of Southampton, Southampton, UK

[5]Centre for Hepatology, Division of Medicine and ULCH-UCL NIHR Biomedical Research Centre, University College London, London, UK

[6]Department of Gastroenterology, Nottingham University Hospitals NHS Trust and Biomedical Research Unit, Nottingham, UK

[7]Medical Research Institute, University of Dundee, Dundee, UK.

For peer review only

Abstract

Objective: To derive and validate a clinical prediction model to estimate the risk of liver disease diagnosis following liver function tests (LFT) and to convert the model to a simplified scoring tool for use in primary care.

Design: Population-based observational cohort study of patients in Tayside Scotland identified as having their LFTs performed in primary care and followed for two years. Biochemistry data was linked to secondary care, prescriptions and mortality data to ascertain baseline characteristics of the derivation cohort. A separate validation cohort was obtained from 19 general practices across the rest of Scotland to externally validate the final model.

***Setting*:** Primary care, Tayside, Scotland

***Participants*:** Derivation cohort: LFT results from 310 511 patients. After exclusions (including: patients under 16 years, patients having initial LFTs measured in secondary care, bilirubin >35 µmol/L, liver complications within 6 weeks and history of a liver condition) derivation cohort contained 95 977 patients with no clinically apparent liver condition. Validation cohort: After exclusions this cohort contained 11 653 patients.

***Primary and secondary outcome measures*:** Diagnosis of a liver condition within two years.

Results: From the derivation cohort (n=95 977), 481 (0.5%) were diagnosed with a liver disease. The model showed good discrimination (C-statistic=0.78). Given low prevalence of liver disease the negative predictive values were high. Positive predictive values were low but rose to 20-30% for high risk patients.

Conclusions: This study successfully developed and validated a clinical prediction model and subsequent scoring tool, the Algorithm for Liver Function Investigations (ALFI), which can predict liver disease risk in patients, with no clinically obvious liver disease, who had their initial LFTs taken in primary care. ALFI can help GPs focus referral on a small subset

For peer review only

of patients with higher predicted risk whilst continuing to address modifiable liver disease risk factors in those at lower risk.

Strengths and limitations of this study

- Our study has successfully developed and validated the first risk prediction model and subsequent user-friendly scoring tool, the Algorithm for Liver Function Investigations (ALFI), for liver condition diagnosis in patients with no obvious liver condition at the time of incident liver function testing in primary care.
- This model can be used to facilitate GP decision making about whom to refer to secondary care.
- GGT was found to be a powerful predictor of liver disease, over and above alkaline phosphatase and transaminase. Since some laboratories do not routinely test for GGT its use should be re-evaluated.
- The observational data lacked some potential predictors of liver disease e.g. alcohol intake and body mass index. However, other available predictors such as LFTs and deprivation may act as surrogate markers for such factors.

For peer review only

Background

GPs commonly request liver function tests (LFTs) but the results rarely identify cases of liver disease [1]. Abnormal liver function tests (LFTs) can be indicators of many different diseases besides those of the liver, such as metastatic malignancy, congestive heart failure and inflammatory or infective conditions [2-5]. It can therefore be difficult for a GP to identify a specific liver disease in patients with abnormal LFTs particularly in those who do not have pathognomonic signs or symptoms. This leads to variation in approaches to further investigation with some patients being inadequately investigated and others at low risk of disease having invasive tests with attendant morbidity and cost [6]. The size of this problem is huge with 22% of patients having their initial panel of LFTs performed in general practice having at least one abnormality in Tayside, Scotland [7]. Referral of all these patients to secondary care would place a large burden on health care system resources, so guidance is needed to ensure that care is focussed on those most in need.

The primary care management of these types of patients might be facilitated through the use of a clinical prediction model which estimates the risk of a specific outcome adjusted for patient characteristics [8]. These models, such as the Framingham cardiovascular disease risk score [9], and FRAX for osteoporotic fracture are regularly used in primary care [10]. It is important that clinical prediction models are assessed for their predictive ability, preferably on an external cohort [11].

This population-based historical cohort study followed-up two separate cohorts of patients living in Scotland with no clinically recognised liver disease who initially had LFTs undertaken in primary care [12]. The aims of the study were:

For peer review only

1. To derive a clinical prediction model using a cohort of patients from Tayside that would estimate the risk of a liver disease over the two years following LFTs taken in primary care;
2. To validate this model geographically and temporally using a different cohort of patients from 19 general practices in Scotland;
3. To convert this model into a user-friendly clinical scoring tool that will allow the GP to easily calculate the risk of liver disease diagnosis at 6 months and 2 years.

Methods

Separate populations were used to develop the prognostic model (derivation cohort) and then validate it (validation cohort).

Derivation cohort

The study population was initially derived from a laboratory database which contains all electronically available LFT results from patients within Tayside during the fifteen year period from January 1989 to December 2003 linked to hospital admission and GP prescribing data [12]. LFTs included bilirubin, albumin, alkaline phosphatase (ALP), gamma-glutamyltransferase (GGT), alanine transaminase and aspartate aminotransferase. Since many laboratories only measure either alanine transaminase or aspartate aminotransferase these two tests were combined as one test and are referred to as transaminases throughout.

Patients aged 16 and above with no obvious or reported clinical signs of a liver disease upon presentation to their GP, with at least 2 different LFTs requested from the index appointment, between 1989 and 2003 were eligible for inclusion. The following exclusion criteria ensured

For peer review only

that the study population of patients had no clinically recognised liver disease at presentation in primary care:

- Bilirubin greater than 35 µmol/L at baseline, suggesting jaundice.
- Diagnosis of ascites, encephalopathy, varices or portal hypertension within 6 weeks of their first LFTs.
- History of any liver disease before baseline.

Databases

The databases relevant to this study are described briefly in Box 1 and in further detail elsewhere [12-18]. In Scotland, all individuals registered with a GP have a unique identifier, the Community Health Index (CHI) [14]. This number is used for all health encounters and is contained within all of the databases used in this study. Since it contains person identifiable information, the CHI was mapped to a project specific pseudonymous ten digit code by the data protection officer at the Health Informatics Centre, University of Dundee, before removing the CHI and releasing the data to the researcher. All of the electronic databases were electronically record-linked deterministically using this project specific pseudonymous code.

Ethics statement

Ethical approval was obtained from the Tayside Committee for Medical Research Ethics in February 2005 (Ref No. 04/S1401/199). Written informed consent from patients was waived by the Tayside Committee for Medical Research Ethics because the databases were anonymised so that no patient identifiable information was accessible. The databases used in this study (see Box 1) covered the entire study period and were used in accordance with

For peer review only

procedures approved under the Caldicott Guardian and the Data Protection Act UK (1998) in line with the European directive of 1995.

Baseline characteristics

As well as the five analysed LFTs, baseline characteristics included age, gender, deprivation [13], comorbidities (including cancer, diabetes, ischaemic heart disease (IHD), stroke, renal disease, respiratory disease and biliary disease), diagnosed alcohol and drug dependency, methadone use, and the use of statins, non-steroidal anti-inflammatory drugs (NSAIDs) or antibiotics in the three months before LFTs. Since patients with bilirubin >35 μmol/L were excluded, bilirubin was categorised into normal and mildly raised (<18 and 18-35 μmol/L respectively for males; <16 and 16-35 μmol/L respectively for females).

Outcome

The primary outcome for this study was liver disease diagnosis during the two years following the initial analysis of LFTs in primary care. Liver diseases were identified from the ELDIT database (Box 1), described in further detail elsewhere [18]. A detailed table of the liver diseases included and their source database is presented in Appendix 1 (available online).

Model derivation

Survival analysis was conducted using parametric regression models to estimate the risk of liver disease within two years. The starting point was taken as the date of the initial LFT test and the endpoint for each patient was whichever one of the following events came first: end of follow-up (i.e. two years later), death, end of study period (i.e. 31st December 2003), date

For peer review only

of emigration, or liver disease diagnosed. All patients whose endpoint was not liver disease diagnosis were censored in the model.

The Weibull regression model was used for model building using potential predictors at baseline. A manual stepwise technique was used to arrive at a model that contained only significant predictive characteristics. The functional form of continuous characteristics (age, albumin, ALP, GGT and transaminase) was assessed by plotting each against the Martingale residuals and, subsequently, appropriate transformations were carried out where necessary. Clinically important two-way predictor interactions were also investigated and were included in the final model if they were statistically significant. The significant predictors from the Weibull model were then refitted to different parametric model distributions including the generalised gamma, log-logistic, log-normal, and exponential distributions to find the one that fitted best [19]. The Akaike's information criterion (AIC) was used to select the optimal model. Covariates which were just outside the significance level for the Weibull model were also added to these other models to check if they became significant. If they did then they were included in that model.

The problem of missing data occurs in almost all retrospective studies using routine health databases. The simplest way of dealing with this is to use only cases with complete data in the analysis. However, this leads to the loss of potentially valuable information from the incomplete cases (and hence loss of power), and introduces bias, especially if there are systematic differences between the complete and incomplete cases. Therefore, the findings from an analysis using only the complete cases may not be a true reflection on what would be found if all the cases were analysed. To analyse only the complete data assumes that the missing data are missing completely at random (MCAR) which is unlikely [20,21]. A weaker

For peer review only

version of the MCAR assumption is the missing at random (MAR) assumption. This differs from MCAR in that it assumes the missing data are dependent on one or more variables in the observed data [21]. Assuming MAR, a multiple imputation technique using the Markov Chain Monte Carlo (MCMC) method was conducted to impute missing values for the LFTs using PROC MI in SAS [22]. Every model was fitted to 30 imputed datasets and the 30 sets of parameter estimates and covariances were combined to produce inferential results using PROC MIANALYZE. All baseline characteristics, time to liver disease (or censored event) and liver disease diagnosis outcome were included in the procedure. The complete data were also analysed separately as a sensitivity analysis.

The integrated discrimination index (IDI) was used to measure the improvement in the final model for each individual covariate [23]. The IDI for a covariate is essentially the difference between the proportion of variance explained by the full model (i.e. adjusted for all covariates) and the model without the covariate of interest. The sensitivity, specificity, PPV, and negative predictive value (NPV) were also calculated for different risk cut-offs, accounting for censoring [23].

Model validation

A separate cohort of patients was obtained from the Primary Care Clinical Informatics Unit (PCCIU), University of Aberdeen [24], and was used to externally validate the final model. The validation cohort contained all patients registered with 19 practices from across Scotland out-with Tayside. The practices were participating in the Practice Team Information project operated by the Information Services Division of the National Health Service National Services Scotland, and contributed data to PCCIU. The patient population within the PCCIU

For peer review only

database is broadly representative of the Scottish population, with respect to age, sex, and social deprivation [25]. The validation cohort contained patients having their initial LFTs measured in primary care between January 2004 and August 2008. All eligible patients had to have test results for ALP, bilirubin, albumin, and transaminase. All baseline characteristics and outcome data obtained for the derivation cohort were also obtained for the validation cohort. The same exclusion criteria listed above was also applied to the validation cohort.

The parameter estimates of the final model from the derivation cohort were applied to the validation cohort to assess its predictive ability. The C-statistic was used as a measure of discrimination [26]. Discrimination assesses the model's ability to correctly distinguish between patients who develop liver disease (for whom the model assigns a high risk) and do not develop liver disease (for whom the model assigns a low risk). The model's predicted probabilities were assessed for accuracy using calibration plots [27]. A calibration slope test was conducted to test for over-fitting of the model and true differences in the effects of predictors [27]. This was done by fitting the linear predictor of the final model in a model by itself and testing its slope. A significant deviation from one signifies over-fitting. The sensitivity, specificity, PPV, and NPV of the model were also calculated for the validation cohort. These assessments were calculated using the validation cohort with the average GGT values across the 30 imputed datasets used for those patients who did not have a GGT measurement.

Decision curve analysis

Decision curve analysis was used to determine a range of threshold predicted probabilities of liver disease where the primary care decision to refer a patient to secondary care would be

For peer review only

better than assuming all patients are disease free (i.e. not referring anyone) and assuming that all patients have liver disease (i.e. referring everyone) [28]. The method involves plotting the net benefit of the model against the threshold probability. The net benefit is defined as the difference between the proportion of patients who are true positive and false positive weighted by the relative harm of a false positive and false negative result. The net benefit of the model is then compared to the scenario where everyone is assumed disease free and therefore not referred (net benefit equals zero) and to the scenario where everyone is assumed disease positive and therefore referred. Threshold probabilities that have a higher net benefit than both of these scenarios means that, for these thresholds, the model is better than referring no one and referring everyone. The analysis was conducted using the validation cohort and the predicted probabilities at the maximum follow-up time of 2 years were used.

Clinical scoring tool

A points-based scoring tool was created from a simpler version of the final model for potential use in primary care [29]. Continuous predictors from the model were dichotomised into clinically and statistically sensible categories to create the tool. Mid-points of the categories were used to estimate the risk of liver disease for each category. The tool estimated short term risk at six months follow-up and longer term risk at two years follow-up.

Model development and validation were performed using SAS (v9.3) (SAS Institute, Cary, North Carolina) and the decision curve analysis was performed using the stdca() function in R version 3.0.1 [30-32].

Results

Baseline characteristics

For peer review only

Before applying the exclusion criteria LFTs were extracted for 310 511 patients. After excluding patients under 16 years old, non-Tayside residents and those who initial LFTs were measured in secondary care, 99 165 patients remained. After excluding those with clinically recognised liver disease at baseline the derivation cohort contained 95 977 patients with incident initial LFTs taken in primary care and with no obvious liver disease. There were more females (57.9%) than males (42.1%), and the median (interquartile range) age was 54.6 (39.2 – 68.8) years (Table 1). The most frequent co-morbidity was IHD (5.6%), followed by cancer (3.8%).

For peer review only

Only 8 388 (8.7%) patients had all five LFTs. The percentage of complete data for each LFT was as follows: ALP (99.2%), albumin (99.2%), bilirubin (93.6%), transaminases (76.5%), and GGT (10.9%). There were more males with complete data (i.e. having all five LFTs) than females (54.6% versus 45.4%) compared to the incomplete data group (40.9% versus 59.1%). The group with complete data were also more deprived and contained more alcohol dependent patients than the incomplete data group (see Appendix 2). When those without GGT measurements were compared to those with GGT measurements the results were similar to the above since those without GGT measurements comprised the majority of the incomplete data group. The group of patients without transaminase measurements contained a higher proportion of females (63.5% versus 36.5%) than that with transaminase measurements (56.2% versus 43.8%) and had a higher median ALP result (86 U/L versus 73 U/L).

Liver disease diagnosis

A total of 481 patients (0.5%) were diagnosed with a liver disease during the two year follow-up period. Of these, 339 (70.5%) had at least one abnormal LFT (out of 22 673). Cirrhosis was the most frequently diagnosed condition (n=75, 15.6%), and in 19 of these the

cause was recorded as alcohol related liver disease. PBC was diagnosed in 73 patients but only as definite in 13 (probable, n=37; possible n=23). The next most frequent diagnoses were Hepatitis C (n=68), alcoholic related liver disease (n=68), Hepatitis B (n=40), hepatocellular carcinoma (n=34), autoimmune hepatitis (n=29) and fatty liver disease (n=28).

Prediction of a liver disease

The log-normal regression model had the lowest AIC and so was proven to have the best fit to the survival distribution. The following baseline characteristics were significant predictors of liver disease diagnosis: increasing GGT, decreasing albumin, alcohol dependency, being female, increasing ALP, living in a deprived area and younger age (Table 2). There were also significant interactions between albumin and transaminase, and albumin and methadone use. NSAID use, antibiotic use, drug dependency, and all comorbidity history indicators were not significantly predictive of a liver disease. The IDI statistic showed that albumin explained the greatest percentage of variance in the model followed by GGT. When the model was fitted to the subgroup of patients with complete data (N=8738), only GGT and albumin were significant out of all the predictors. However, the parameter estimates had the same direction of effect and reasonably similar size of effect. The added heterogeneity from the subgroup of patients with imputed GGT and the increased power from the much larger study population would explain the significance of all the predictors in the imputed model.

Validation

For peer review only

After exclusions, the external cohort contained 11 653 patients with incident initial LFTs taken in primary care. A total of 57 patients (0.5%) were diagnosed with a liver disease within two years. The proportion of males and females were reasonably similar to the derivation cohort (45.2% versus 42.1% males), but the external cohort was older, more deprived and had a greater proportion of co-morbidities and medications (Table 1). However, the median LFTs were similar between the two cohorts. GGT was missing for 4178 (35.9%) patients and was imputed using the same method as for the derivation cohort. The average GGT measure for each patient from the 30 imputed datasets was used where the original measure was missing so that the validation could be performed in one dataset.

The C-statistic for the final model parameter estimates applied to the external cohort was 0.78 (95% CI 0.72 to 0.84). The calibration plot (Appendix 3) showed some visible evidence of over-fitting for the highest risk group, however the calibration slope showed no significant deviation from 1 (slope = 0.871 (95% CI 0.696 to 1.047); p=0.15).

The sensitivity, specificity, PPV and NPV for different cut-offs of predicted risk of liver disease diagnosis along with the receiver operating characteristic curves for the final model applied to the derivation and external validation cohorts are displayed in Appendix 4. A cut-off greater than or equal to the 75th percentile of the risk score (i.e. 0.43% in the derivation cohort and 0.57% in the validation cohort) had sensitivity and specificity of approximately 75% each. The NPV was very high at almost 100% for all cut-offs. Although PPV was very low it rose to over 10% for predicted risks over 5% and reached 20-30% for higher risks (over 20%). These performance measures were similar for both derivation and validation cohorts. From the decision curve in Figure 1 it can be seen that the range of threshold

For peer review only

predicted probabilities where the prediction model is of value is approximately between 0.5% and 7.5%.

Predicted probabilities

The final model and how to calculate the risk of liver disease directly from it is presented in Appendix 5. Figure 2 shows the risk of diagnosis over two years for the average risk patient and two example patients with different characteristics as described in the legend.

Clinical scoring tool

A user friendly paper-based scoring tool was developed from a slightly simpler version of the final model with the albumin and transaminase interaction term removed (Figure 3). The C-statistic for the scoring tool when applied to the validation cohort was 0.77 (95% CI 0.59 to 0.89). When the tool's approximation to the linear predictor was used in a calibration slope test it was not significantly different from one, Beta=0.93 (0.73 to 1.14), p=0.53. The scores for each of the risk factors add up to a total score that corresponds to the risk of liver disease. The baseline case for the tool was a 45 year old female, living in an affluent area, who was not alcohol or methadone dependent with transaminase=11.5U/L, ALP=46 U/L, GGT=17 U/L and albumin=48g/L. Her score of zero corresponded to a liver disease risk of 0.03%. As an example, a 60 year old male living in a deprived area who is alcohol dependent, but not a methadone user, with transaminase=70U/L, ALP=130U/L, GGT=45U/L and albumin=38 has a score of 29 which corresponds to a liver disease risk within two years of 4.1%. For comparison, when this patient's characteristics were entered into the full model in appendix 5 their risk was 3.8%.

For peer review only

The correlation between the predicted probabilities from the scoring tool probabilities and the predicted probabilities from the final model when applied to the validation cohort was 0.91. Appendix 6 shows the scatterplot of the relationship between the two.

Discussion

Summary

A user-friendly clinical scoring tool has been developed that can estimate the risk of liver disease diagnosis at six months and two years in patients who had their initial LFTs measured in primary care. The tool is based on a clinical prediction model which was derived and validated using large population databases. This model adds morbidity and a longer duration to a previous algorithm that predicts one year mortality as part of our Algorithms for Liver Function Investigations (ALFI) [33].

Strengths and limitations

This is the first large-scale population-based analysis of LFTs with complete determination of liver disease outcome. ALFI has been derived from unselected "real-world" observations in a geographically defined population: an approach recommended by the National Institutes of Health [34]. A weakness of the electronic databases was the lack of some potential predictors of liver disease e.g. alcohol intake and body mass index, which the GP can readily ascertain to guide decisions. However, predictors in the model such as LFTs and deprivation may act as surrogate markers for such factors. Whilst we have no data on the clinical indications for LFTs being measured, we were able to identify patients with a history of major co-morbidities since 1980, including cancer, diabetes and IHD using hospital admission records and population registers, and patients who were prescribed statins, NSAIDs, and antibiotics. Using the predictors we did have, the model was proven to have good ability to predict liver

For peer review only

disease. There will have been underascertainment of some liver diseases; particularly those diagnosed in primary care, such as mild alcohol related liver disease and the steatotic end of the spectrum of non-alcoholic fatty liver disease. Patients with these diseases are most likely to be dealt with by being advised to lose weight or reduce alcohol intake, and only those who developed further problems and who were referred would be captured on the database.

The aim of the study was to combine known significant predictors of liver disease into one source of prognostic information that could provide the GP with an individualised estimate of the predicted risk of liver disease at the point where the patient has had their initial LFTs measured. If, at that point, the patient has a high risk of liver disease based on this model then the GP can also use other contextual information and their own clinical judgement to make a decision as to whether to monitor or refer the patient to a liver clinic according to national or local protocols.

The predicted probabilities from the scoring tool correlated well with those from the model. However, it is evident from the scatterplot in Appendix 6 that for high predicted probabilities from the final model the tool under-estimates the predicted probability. This is because many of these higher probabilities come from patients who have very highly abnormal LFTs. The tool assigns such patients the points based on the highest cut-off in the tool. For example, if a patient has an ALP measure of 825 the tool assigns the same points as someone with an ALP of 181 (i.e. +4 points) since >=180U/L is the highest cut-off in the tool. It would be inefficient to expand the number of groups for the LFTs to include such large values as the score card would be huge. In such circumstances the GP would automatically refer without the need for a scoring tool. Also, it is advisable to reduce the influence of extreme values

For peer review only

when creating such a scoring tool by using the 99th percentile value of measurements as the largest possible value when creating the cut-offs [29].

External validation

The validation cohort came from 19 general practices across nine different regions of Scotland during a different time period than the derivation cohort. Good discrimination and calibration demonstrated that ALFI is transportable to different geographical and temporal populations. The C-statistic for discriminatory ability was 0.78 which is comparable to that found in the validation of the Framingham equation on different cohorts ranging from 0.63 to 0.83 [35]. The external cohort also contained more deprived and comorbid patients than the derivation cohort proving that ALFI is also robust to changes in baseline characteristics.

GGT and transaminase

GGT and, to a lesser extent, transaminase were either missing or not tested for a large proportion of patients in the derivation cohort but much less so in the validation cohort. From the database, it was not possible to determine the exact reason for the non-presence of these two LFTs. However, in Tayside, the laboratories do not routinely include GGT with the other four LFT results unless specifically requested by the primary care physician. The demographics of the patients with complete data (i.e. males, illicit drug users, alcohol dependents, and patients living in deprived areas) suggested that some primary care physicians may have requested GGT where they suspected substance abuse.[7] Testing bone biochemistry may explain the reason for transaminase not being measured since this group contained a higher proportion of females who are more susceptible to bone disease, such as osteoporosis, and had a higher median ALP which is a marker for bone disease. Furthermore, one of the hospitals in Tayside analysed transaminase using a separate analyser for several

For peer review only

years throughout the study and did not keep electronic copies which may also explain some of the missing values. Therefore it was assumed that the missing/untested LFT data depended on variables in the observed data, the assumption required for multiple imputation, [22] and the appropriate guidelines for handling this problem were followed [36].

Implications for practice and research

At a cut-off of 0.6% (the 75th percentile) for liver disease risk, sensitivity and specificity were similar with values of 73.7% and 75.3% in the validation cohort. However, the PPV for low cut-offs such as this was poor. The specificity at a cut-off of 1.2% risk is greater than 90% and rises to 100% at the 10% cut-off. The NPV of the model are very high even for reasonably small cut-offs of risk meaning that the model is good at ruling out risk of liver disease within two years. However, this is not surprising given the low prevalence of liver disease in the sample of 0.5% [37]. The PPV was low since liver disease diagnosis was relatively rare but it rose to over 10% for predicted risks of over 5% and in a small minority of patients with higher predicted risk reached 20-30% with the NPV remaining very high. Therefore ALFI may be most accurate at higher predicted risk cut-offs with reasonable PPVs. As well as displaying reasonable accuracy, it is also important to show that the model has the potential to improve decision making.

The decision curve analysis showed that the model was of value for threshold probabilities between 0.5% and 7.5%. To determine whether the model is of clinical value, we should consider the possible range of threshold probabilities of liver disease at which GPs would decide to refer the patient to secondary care. The decision to refer is not a particularly risky intervention for the patient. Initially, it will lead to further blood tests and possible

For peer review only

ultrasound in secondary care. The outcome of these results will determine whether the patient requires a liver biopsy which has some risk attached to it. However, if the patient gets to that stage then the clinician must have a high suspicion of liver disease, meaning that the decision to refer was correct. If the GP does not refer and the patient does have or develop liver disease, then the risk to the patient (i.e. of being a false negative) depends on the type and stage of the disease. Therefore, the GP may think that the intervention of referral is much less risky than not referring and decide that a probability threshold of, say, 5% is enough to refer. For thresholds between 5-7.5% the model is also reasonably accurate. Therefore ALFI could help GPs focus referral on a small subset of patients with higher predicted risk and reasonable PPVs whilst taking clinical factors that were not included in the model (but which may have improved prediction) into account. However, where the risk is lower, the long timeframe of the development of many liver diseases means that GPs can continue to address modifiable liver disease risk factors (e.g. alcohol misuse). . The next step is to evaluate ALFI as a complex intervention whilst taking into account the cost associated with referral of false positives [38].

GGT should be considered just as important as other LFTs in the prediction of liver disease since only albumin explained more variation in the model. Proper use of ALFI in practice will depend on GGT being a routine part of the LFT panel. Local health economies will have to decide if the additional cost of an extra test in the panel is worth it in terms of GGT's value in improving the ability to predict those patients who at high (or low) risk and, subsequently, change referral practice. Before such decisions are made further research is necessary involving cost-effectiveness analysis.

For peer review only

*

Conclusions

In summary, this study has developed and externally validated the ALFI model for prediction of liver disease diagnosis in patients with no clinically obvious liver disease having their LFTs taken within primary care. From this model, a simple scoring tool was developed to facilitate GP decision making with regards to retesting or referring their patients. GP decisions regarding referral of patients to secondary care should be based on probability thresholds where benefits outweigh harm [39]. ALFI requires further evaluation as a complex intervention but it has the potential to save health service costs and prevent unnecessary further investigations and treatments.

For peer review only

Acknowledgements

The authors wish to thank Alison Bell from HIC for extracting and pseudonymising all the datasets used. We thank Primary Care Clinical Informatics Unit (PCCIU) at the University of Aberdeen for providing the Practice Team Initiative cohort for external validation of our model.

Funding

This work was supported by the UK National Health Service Research & Development Programme Health Technology Assessment Programme (project number 03/38/02) and supported by the Backett Weir Russell Career Development Fellowship.

Department of Health Disclaimer: The views and opinions expressed therein are those of the authors and do not necessarily reflect those of the Department of Health.

The study funder did not participate in the study design; in the collection, analysis and interpretation of data; in the writing of the report; or in the decision to submit the article for publication.

Authors' contributions

DJM contributed to the design of the study, conducted the analyses, drafted and finished the paper. JFD, FMS, PR, WR and SR contributed to the design and conduct of the study, and the writing of the paper. PTD contributed to, and oversaw the design and conduct of the study, and contributed to the writing of the paper. He is the guarantor for the paper. All authors read and approved the final manuscript.

Competing interests

None declared.

Data Sharing Statement

No additional data available

For peer review only

References

1. Pratt DS, Kaplan MM: Evaluation of abnormal liver-enzyme results in asymptomatic patients. *N Engl J Med* 2000, 342:1266-1271.

2. Schalk BWM, Visser M, Bremmer MA, et al.Change of serum albumin and risk of cardiovascular disease and all-cause mortality: Longitudinal Aging Study Amsterdam. *Am J Epidemiol* 2006, 164:969-977.

3. Sorensen HT, Moller-Petersen JF, Felding P, Andreasen C, Nielsen JO: Epidemiology of abnormal liver function tests in general practice in a defined population in Denmark. *Dan Med Bull* 1991, 38:420-422.

4. Kim HC, Nam CM, Jee SH, et al. Normal serum aminotransferase concentration and risk of mortality from liver diseases: Prospective cohort study. *Br Med J* 2004, 328:983-986.

5. Roderick P: Commentary: Liver function tests: defining what's normal. *Br Med J* 2004, 328:987.

6. Sherwood P, Lyburn I, Brown S, et al. How are abnormal results for liver function tests dealt with in primary care? Audit of yield and impact. *Br Med J* 2001, 322:276-278.

7. McLernon DJ, Donnan PT, Ryder S, et al.Health outcomes following liver function testing in primary care: a retrospective cohort study. *Fam Pract* 2009, 26:251-259.

8. Moons K, Royston P, Vergouwe Y, et al. Prognosis and prognostic research: what, why, and how? *Br Med J* 2009, 338:b375.

9. Fornasini M, Brotons C, Sellarès J, et al. Consequences of using different methods to assess cardiovascular risk in primary care. *Fam Pract* 2006, 23:28-33.

10. Siris ES, Baim S, Nattiv A: Primary care use of FRAX: absolute fracture risk assessment in postmenopausal women and older men. *Postgrad Med* 2010, 122:82-90.

11. Altman DG, Vergouwe Y, Royston P, et al.Prognosis and prognostic research: validating a prognostic model. *Br Med J* 2009, 338:b605.

For peer review only

12. Donnan PT, McLernon D, Dillon JF, et al. Development of a decision support tool for primary care management of patients with abnormal liver function tests without clinically apparent liver disease: a record-linkage population cohort study and decision analysis (ALFIE). *Health Technol Assess* 2009, 13(25).

13. Carstairs V, Morris R: Deprivation and mortality: an alternative to social class? *Community Med* 1989, 11:210-219.

14. Evans JMM, MacDonald TM: Record-linkage for pharmacovigilance in Scotland. *Br J Clin Pharm* 1999, 47:105-110.

15. NHS National Services Scotland: SMR01 – General/Acute Inpatient and Day Case [http://www.datadictionary.scot.nhs.uk/isddd/9065.html]

16. Morris AD, Boyle DI, MacAlpine R, et al. The diabetes audit and research in Tayside Scotland (darts) study: electronic record linkage to create a diabetes register. *Br Med J* 1997, 15:524-528.

17. Donnan PT, Wei L, Steinke DT, et al. Presence of bacteriuria caused by trimethoprium resistant bacteria in patients prescribed antibiotics: multilevel model with practice and individual patient data. *Br Med J* 2004, 328:1297-1301.

18. Steinke DT, Weston TL, Morris AD, et al. : The epidemiology of liver disease in Tayside database: a population-based record-linkage study. *J Biomed Inform* 2003, 35:186-193.

19. Collett D. *Modelling survival data in medical research*. 2 ed. Boca Raton: Chapman & Hall/CRC, 2003.

20. Rubin DB: Inference and missing data. *Biometrika* 1976, 63:581–592.

21. Rubin DB: *Multiple imputation for nonresponse in surveys*. New York: Wiley; 1987.

22. Yuan YC: Multiple imputation for missing data: concepts and new development: SAS Institute Inc. [http://support.sas.com/rnd/app/papers/multipleimputation.pdf].

For peer review only

23. Chambless LE, Cummiskey CP, Cui G: Several methods to assess improvement in risk prediction models: extension to survival analysis. *Stat Med* 2011, 30:22-38.

24. University of Aberdeen: Primary Care Clinical Informatics Unit [http://www.abdn.ac.uk/pcciu/index.htm]

25. Information Services Division Scotland: General Practice – Practice Team Information (PTI) [http://www.isdscotland.org/isd/1283.html#Background_to_PTI]

26. Pencina MJ, D'Agostino RB: Overall C as a measure of discrimination in survival analysis: model specific population value and confidence interval estimation. *Stat Med* 2004, 23:2109-2123.

27. Steyerberg EW: *Clinical prediction models: a practical approach to development, validation, and updating*. New York: Springer; 2009.

28. Vickers AJ, Elkin EB: Decision curve analysis: a novel method for evaluating prediction models. *Med Decis Making* 2006, 26: 565.29. Sullivan LM, Massaro JM, D'Agostino Sr RB: Tutorial in biostatistics: Presentation of multivariate data for clinical use: The Framingham Study risk score functions. *Stat Med* 2004, 23:1631-1660.

30. R Core Team. R: A language and environment for statistical computing. R Foundation for Statistical Computing, Vienna, Australia 2014 [http://www.R-project.org].

31. Vickers AJ, Memorial Sloan Kettering Cancer Centre: Epidemiology and Biostatistics: Decision curve analysis. [http://www.mskcc.org/research/epidemiology-biostatistics/health-outcomes/decision-curve-analysis-0].32. Vickers AJ, Cronin AM, Elkin EB, Gonen M: Extensions to decision curve analysis, a novel method for evaluating diagnostic tests, prediction models and molecular markers. *BMC Med Inform Decis Mak*, 8:53.

33. McLernon DJ, Dillon JF, Sullivan FM, et al: The utility of liver function tests for mortality prediction within one year in primary care using the Algorithm for Liver Function Investigations (ALFI). *PLoS ONE* 2012, 7:e50965.

For peer review only

34. Dorans K: Pilot projects aim to ease access to clinical data. *Nature Med* 2009, 15:226.

35. D'Agostino RB, Grundy S, Sullivan LM, et al: Validation of the Framingham Coronary Heart Disease Prediction Scores. Results of a multiple ethic groups investigation. *JAMA* 2001, 286:180-187.

36. Sterne JAC, White IR, Carlin JB, et al: Multiple imputation for missing data in epidemiological and clinical research: potential and pitfalls. *Br Med J* 2009, 338:b2393.37. Hawkins RC. The evidence based medicine approach to diagnostic testing: practicalities and limitations. *Clin Biochem Rev* 2005, 26:7-18.

38. Craig P, Dieppe P, Macintyre S, et al. Developing and evaluating complex interventions: the new Medical Research Council guidance. *Br Med J* 2008, 337:a1655.

39. Goodwin N. Diagnostic delays and referral management schemes: how 'integrated' primary care might damage your health. *Int J Integr Care* 2008, 8:e78.

For peer review only

Box 1: Databases record-linked to create the Tayside derivation cohort

1. Death registry from the General Register Office for Scotland including date and causes of death.
2. Deprivation score was assessed by the Carstairs method [13]. The score was divided into two categories of affluent and deprived.
3. Tayside Community Health Index (CHI) files. Contains CHI number [14], name, address and date of birth for all individuals registered with a general practitioner in Tayside. It also holds migration dates to and from Tayside. The database was pseudonymised before release to the researcher.
4. Regional biochemistry database. All LFTs came from the largest hospital laboratory in the Tayside region, Ninewells. Two smaller hospital laboratories contributed electronic LFT results later, one from 1998 and the other from 2003.
5. Scottish Morbidity Records 1 (SMR01) database: Patient specific morbidity data are routinely collected in Scotland and collectively these are known as Scottish Morbidity Records (SMR). SMR01 contains hospital admissions and procedures for all hospitals in Scotland and is one of the oldest and most complete national health datasets in the world [15]. Major co-morbidity groups at baseline were identified using this database, and the Diabetes Audit and Research Tayside Scotland (DARTS) and Heart disease Evidence-based Audit and Research Tayside Scotland (HEARTS) databases for diabetes and chronic heart disease respectively [16].
6. SMR04 database: Holds all inpatient and day case episodes for mental health specialties, which identified patients admitted/discharged with diagnoses of alcohol dependency or drug misuse.
7. Pharmacist dispensed prescription database: A community based database that holds encashed prescriptions in Tayside [17], and allowed identification of patients on some potentially hepatotoxic drugs including statins, antibiotics, and Non-Steroidal Anti-Infammatory Drugs (NSAIDs).
8. Epidemiology of Liver Disease In Tayside (ELDIT) database: Contains all Tayside patients with liver diseases that have been ‘electronically’ diagnosed using record-linkage of biomedical datasets, including virology and immunology [18]. ELDIT was used for the study exclusion criteria (i.e. previous liver disease, liver disease complications within 6 weeks) and for the outcome of liver disease diagnosis during the two-year follow-up period.

Table 1 Baseline characteristics of patients in the derivation (n=95977) and validation (n=11653) cohorts

Baseline Characteristics		Cohort n(%) *or* Median (IQR)	
		Derivation	Validation
Age (years)		54.6 (39.2-68.8)	60.0 (47.0, 72.0)
Gender	Male	40374 (42.1)	5271 (45.2)
	Female	55603 (57.9)	6382 (54.8)
Carstairs category[1]	Affluent	47286 (49.3)	2753 (23.6)
	Deprived	48691 (50.7)	8900 (76.4)
Comorbidity history	Cancer[2]	3629 (3.8)	956 (8.2)
	Diabetes	1386 (1.4)	1441 (12.4)
	IHD	5370 (5.6)	2034 (17.5)
	Renal disease	141 (0.2)	155 (1.3)
	Respiratory disease	2636 (2.8)	883 (7.6)
	Stroke	1471 (1.5)	583 (5.0)
Medication in previous 3 months	Statins	3176 (3.3)	3178 (27.3)
	NSAIDs	6698 (7.0)	1762 (15.1)
	Antibiotics	8307 (8.7)	1962 (16.8)
Abusive substance	Alcohol	2632 (2.7)	465 (4.0)
	Drug	371 (0.4)	0 (0.0)
	Methadone	377 (0.4)	10 (0.1)
Liver function tests	Albumin (g/L)	44.0 (42.0-46.0)	44.0 (41.0, 46.0)
	ALP (U/L)	76.0 (62.0-94.0)	75.0 (62.0, 92.0)
	Transaminase (U/L)	18.0 (14.0-26.0)	21.0 (16.0, 30.0)
	GGT (U/L)	26.0 (17.0-47.0)	27.0 (18.0, 45.0)
	Bilirubin[3]		
	Normal	81111 (91.0)	10587 (90.8)
	Mildly raised	8058 (9.0)	1066 (9.2)

Data reported are median (interquartile range) or percentage

For peer review only

[1]Carstairs categories 1 to 3 were recoded as affluent and categories 4 to 7 were recoded as deprived.

[2]Not including biliary cancer or hepatocellular cancer;

[3]Normal bilirubin defined as 0-15□mol/L for females, 0-17μmol/L for males; Mildly raised bilirubin defined as 16-35 μ mol/L for females, 18-35 μ mol/L for males.

IQR interquartile range; IHD ischaemic heart disease; NSAID non-steroidal anti-inflammatory; ALP alkaline phosphatase; GGT gamma-glutamyltransferase.

For peer review only

Table 2 Parameter estimates (95% CI) and IDI for the final log-normal regression model predicting risk of a liver disease diagnosis within 2 years of initial liver function tests (481 diagnosed)

Parameter	Coefficient (95% CI)	P-value	IDI, %
Intercept	9.524 (1.986 to 17.062)	0.013	
Albumin	0.488 (0.306 to 0.669)	<0.001	0.711[a]
Log(GGT)	-1.704 (-2.223 to -1.184)	<0.001	0.689
Methadone (yes *v* no)	8.319 (-2.019 to 18.657)	0.115	0.465[a]
Log(ALP)	-0.739 (-1.213 to -0.264)	0.002	0.307
Log(transaminase)	2.016 (-0.151 to 4.183)	0.068	0.266[a]
Alcohol dependent (yes *v* no)	-1.210 (-1.856 to -0.563)	<0.001	0.143
Gender (Male *v* Female)	0.583 (0.236 to 0.930)	0.001	0.135
Age at baseline	0.014 (0.004 to 0.024)	0.009	0.121
Deprived[b] (yes *v* no)	-0.518 (-0.852 to -0.183)	0.002	0.013
Methadone X Albumin	-0.295 (-0.530 to -0.061)	0.014	
Albumin X Log(transaminase)	-0.070 (-0.122 to -0.018)	0.008	
Scale	4.551 (4.192 to 4.910)	<0.001	

GGT = gamma-glutamyltransferase; ALP = alkaline phosphatase; IDI=integrated discrimination improvement.

[a] the relative interaction terms containing this parameter were also excluded.

[b] Carstairs categories 1 to 3 were coded as affluent and categories 4 to 7 were coded as deprived.

Note: Parameter estimates are in decreasing order of IDI. A negative coefficient indicates an increased risk of diagnosis, whilst a positive coefficient indicates a decreased risk of diagnosis. However, this differs for terms involved in interactions i.e. increasing transaminase increases risk; for a methadone user, increasing albumin increases risk; for non-methadone users, decreasing albumin increases risk.

For peer review only

Figure titles and legends

Figure 1. Decision curve for a model to predict liver disease diagnosis in patients having their LFTs measured in primary care.

Dashed line: Prediction model; grey line: assume all patients have liver disease; black line: assume no patients have liver disease.

Figure 2. Probability of a liver disease diagnosis during the two years for the average risk patient and two example patients with different risk levels.

Note: Average risk is defined as the risk calculated using the average linear predictor value from the model. Patient 1 and Patient 2 characteristics are presented in the boxes within the plot. The estimated risk of being diagnosed with a liver disease within two years for each of these patients is 0.002 (average risk patient), 0.097 (Patient 1) and 0.493 (Patient 2).

Figure 3. Clinical scoring tool for likely liver disease diagnosis in primary care.

For each risk factor category enter the corresponding score into the box on the right hand side. Sum the scores in the total score box. Look for the total score in the lower table and read off the risk of liver disease within 6 months and/or 2 years.

For peer review only

List of Supplementary Materials

File name: Appendix 1

File format: .docx

Title of data: Liver diseases from the ELDIT database

Description of data: Liver diseases from the ELDIT database used as the outcome in the model and comorbidities used as potential predictors of the outcome along with their original clinical database source(s)

File name: Appendix 2

File format: .doc

Title of data: Complete case analyses

Description of data: Comparison of baseline characteristics in patients with complete data for all five LFTs with patients with incomplete data. Model fitted to complete data only.

File name: Appendix 3

File format: .docx

Title of data: Calibration plots

Description of data: Calibration plots for the final model applied to the validation cohort

File name: Appendix 4

File format: .docx

Title of data: Performance of the final model to predict liver disease diagnosis

Description of data: Receiver operating characteristic (ROC) curves for the final model to predict liver disease diagnosis applied to the validation cohort. Sensitivity, specificity,

For peer review only

positive predictive value (PPV), and negative predictive value (NPV) for different percentiles of liver disease risk for the derivation and validation cohorts are also presented.

File name: Appendix 5

File format: .docx

Title of data: Calculating the risk of liver disease diagnosis within two years from the final model.

Description of data: A mathematical formula showing how to calculate the predicted risk of liver disease diagnosis at any time point during a two year maximum follow-up period. A worked example is also presented.

File name: Appendix 6

File format: .docx

Title of data: Scatterplot of the predicted probability of liver disease diagnosis from the full model against the predicted probability from the scoring tool.

Description of data: Scatterplot to show the correlation between the predicted probability of liver disease diagnosis from the full model and the predicted probability of liver disease diagnosis from the scoring tool.

For peer review only

Prediction of liver disease in patients whose liver function tests have been checked in primary care: model development and validation using population-based observational cohorts

David J McLernon[1*]
* Corresponding author
Email: d.mclernon@abdn.ac.uk

Peter T Donnan[2]
Email: p.t.donnan@dundee.ac.uk

Frank M Sullivan[3]
Email: ~~f.m.sullivan@dundee.ac.uk~~frank.sullivan@nygh.on.ca

Paul Roderick[4]
Email: pjr@soton.ac.uk

William M Rosenberg[5]
Email: w.rosenberg@ucl.ac.uk

Steve D Ryder[6]
Email: Stephen.Ryder@nuh.nhs.uk

John F Dillon[7]
Email: j.dillon@nhs.net

[1]Medical Statistics Team, Division of Applied Health Sciences, College of Life Sciences and Medicine, University of Aberdeen, UK

[2]Dundee Epidemiology and Biostatistics Unit, Division of Population Health Sciences, Medical Research Institute, University of Dundee, Dundee, UK

[3]Department of Community and Family Medicine, University of Toronto, Canada~~Division of Population Health Sciences, Medical Research Institute, University of Dundee, Dundee, UK~~

[4]Academic Unit of Primary Care and Population Sciences, Faculty of Medicine, University of Southampton, Southampton, UK

[5]Centre for Hepatology, Division of Medicine and ULCH-UCL NIHR Biomedical Research Centre, University College London, London, UK

[6]Department of Gastroenterology, Nottingham University Hospitals NHS Trust and Biomedical Research Unit, Nottingham, UK

[7]Medical Research Institute, University of Dundee, Dundee, UK.

For peer review only

Abstract

Objective: To derive and validate a clinical prediction model to estimate the risk of liver disease diagnosis following an abnormal liver function tests (LFT) result and to convert the model to a simplified scoring tool for use in primary care.

Design: Population-based observational cohort study of patients in Tayside Scotland identified as having their LFTs performed in primary care and followed for two years. Biochemistry data was linked to secondary care, prescriptions and mortality data to ascertain baseline characteristics of the derivation cohort. A separate validation cohort was obtained from 19 general practices across the rest of Scotland to externally validate the final model.

Setting: Primary care, Tayside, Scotland

Participants: Derivation cohort: LFT results from 310 511 patients. After exclusions (including: patients under 16 years, patients having initial abnormal LFTs measured in secondary care, bilirubin >35 µmol/L, liver complications within 6 weeks and history of a liver condition) derivation cohort contained 95 977 patients with no clinically apparent liver condition. Validation cohort: After exclusions this cohort contained 11 653 patients.

Primary and secondary outcome measures: Diagnosis of a liver condition within two years.

Results: From the derivation cohort (n=95 977), 481 (0.5%) were diagnosed with a liver disease. The model showed good discrimination in both cohorts (C-statistic=0.78). A predicted risk <5% had good ability to 'rule out' liver disease (specificity=99.4%, negative predictive value=99.5%).Given low prevalence of liver disease the negative predictive values were high. Positive predictive values were low but rose to 20-30% for high risk patients.

Conclusions: This study successfully developed and validated a clinical prediction model and subsequent scoring tool, the Algorithm for Liver Function Investigations (ALFI), that), which can predict liver disease risk in patients, with no clinically obvious liver disease, who had their initial LFTs taken in primary care. ALFI can help GPs focus referral on a small subset

For peer review only

of patients with higher predicted risk whilst continuing to address modifiable liver disease risk factors in those at lower risk.GPs who use ALFI to support their decision-making process can reassure patients at low risk. Clinical review of low risk patients instead of further investigations has the potential to reduce iatrogenic disease and save health service costs.

Strengths and limitations of this study

- Our study has successfully developed and validated the first risk prediction model and subsequent user-friendly scoring tool, the Algorithm for Liver Function Investigations (ALFI), for liver condition diagnosis in patients with no obvious liver condition at the time of incident liver function testing in primary care.
- This model can be used to facilitate GP decision making with regardsabout whom to referral to secondary care.
- GGT was found to be a powerful predictor of liver disease, over and above alkaline phosphatase and transaminase. Since some laboratories do not routinely test for GGT its use should be re-evaluated.
- The observational data lacked some potential predictors of liver disease e.g. alcohol intake and body mass index. However, other available predictors such as LFTs and deprivation may act as surrogate markers for such factors.

For peer review only

Background

GPs commonly request liver function tests (LFTs) but the results rarely identify cases of liver disease [1]. Abnormal liver function tests (LFTs) can be indicators of many different diseases besides those of the liver, such as metastatic malignancy, congestive heart failure and ~~systemic~~ inflammatory or infective conditions ~~such as viral infections~~ [2-5]. It can therefore be difficult for a GP to identify a specific liver disease in patients with abnormal LFTs particularly in those who do not have pathognomonic signs or symptoms. This leads to variation in approaches to further investigation with some patients being inadequately investigated and others at low risk of disease having invasive tests with attendant morbidity ~~or mortality~~and cost [6]. The size of this problem is huge with 22% of patients having their initial panel of LFTs performed in general practice having at least one abnormality in Tayside, Scotland [7]. Referral of all these patients to secondary care would place a large burden on health care system resources, so guidance is needed to ensure that care is focussed on those most in need.

The primary care management of these types of patients might be facilitated through the use of a clinical prediction model which estimates the risk of a specific outcome adjusted for patient characteristics [8]. These models, such as the Framingham cardiovascular disease risk score [9], and FRAX for osteoporotic fracture are regularly used in primary care [10]. It is important that clinical prediction models are assessed for their predictive ability, preferably on an external cohort [11].

This population-based historical cohort study followed-up two separate cohorts of patients living in Scotland with no clinically recognised liver disease who initially had LFTs undertaken in primary care [12]. The aims of the study were:

For peer review only

1. To derive a clinical prediction model using a cohort of patients from Tayside that would estimate the risk of a liver disease over the two years following LFTs taken in primary care;
2. To validate this model geographically and temporally using a different cohort of patients from 19 general practices in Scotland;
3. To convert this model into a user-friendly clinical scoring tool that will allow the GP to easily calculate the risk of liver disease diagnosis at 6 months and 2 years.

For peer review only

Methods

Separate populations were used to develop the prognostic model (derivation cohort) and then validate it (validation cohort).

Derivation cohort

The study population was initially derived from a laboratory database which contains all electronically available LFT results from patients within Tayside during the fifteen year period from January 1989 to December 2003 linked to hospital admission and GP prescribing data [12]. LFTs included bilirubin, albumin, alkaline phosphatase (ALP), gamma-glutamyltransferase (GGT), alanine transaminase and aspartate aminotransferase. Since many laboratories only measure either alanine transaminase or aspartate aminotransferase these two tests were combined as one test and are referred to as transaminases throughout.

Patients aged 16 and above with no obvious or reported clinical signs of a liver disease upon presentation to their GP, with at least 2 different LFTs requested from the index appointment, between 1989 and 2003 were eligible for inclusion. The following exclusion criteria ensured

that the study population of patients had no clinically recognised liver disease at presentation in primary care:

- Bilirubin greater than 35 µmol/L at baseline, suggesting jaundice.
- Diagnosis of ascites, encephalopathy, varices or portal hypertension within 6 weeks of their first LFTs.
- History of any liver disease before baseline.

Databases

The databases relevant to this study are described briefly in Box 1 and in further detail elsewhere [12-18]. In Scotland, all individuals registered with a GP have a unique identifier, the Community Health Index (CHI) [14]. This number is used for all health encounters and is contained within all of the databases used in this study. Since it contains person identifiable information, the CHI was mapped to a project specific pseudonymous ten digit code by the data protection officer at the Health Informatics Centre, University of Dundee, before removing the CHI and releasing the data to the researcher. All of the electronic databases were electronically record-linked deterministically using this project specific pseudonymous code.

Ethics statement

Ethical approval was obtained from the Tayside Committee for Medical Research Ethics in February 2005 (Ref No. 04/S1401/199). Written informed consent from patients was waived by the Tayside Committee for Medical Research Ethics because the databases were anonymised so that no patient identifiable information was accessible. The databases used in this study (see Box 1) covered the entire study period and were used in accordance with

For peer review only

procedures approved under the Caldicott Guardian and the Data Protection Act UK (1998) in line with the European directive of 1995.

Baseline characteristics

As well as the five analysed LFTs, baseline characteristics included age, gender, deprivation [13], comorbidities (including cancer, diabetes, ischaemic heart disease (IHD), stroke, renal disease, respiratory disease and biliary disease), diagnosed alcohol and drug dependency, methadone use, and the use of statins, non-steroidal anti-inflammatory drugs (NSAIDs) or antibiotics in the three months before LFTs. Since patients with bilirubin >35 µmol/L were excluded, bilirubin was categorised into normal and mildly raised (<18 and 18-35 µmol/L respectively for males; <16 and 16-35 µmol/L respectively for females).

Outcome

The primary outcome for this study was liver disease diagnosis during the two years following the initial analysis of LFTs in primary care. Liver diseases were identified from the ELDIT database (Box 1), described in further detail elsewhere [18]. A detailed table of the liver diseases included and their source database is presented in Appendix 1 (available online).

Model derivation

Survival analysis was conducted using ~~a~~ parametric regression models to estimate the risk of liver disease within two years. The starting point was taken as the date of the initial LFT test and the endpoint for each patient was whichever one of the following events came first: end of follow-up (i.e. two years later), death, end of study period (i.e. 31st December 2003), date

For peer review only - http://bmjopen.bmj.com/site/about/guidelines.xhtml

of emigration, or liver disease diagnosed. All patients whose endpoint was not liver disease diagnosis were censored in the model.

The Weibull regression model was used for model building using ~~adjusted for the~~ potential predictors at baseline. A manual stepwise technique was used to arrive at a model that contained only significant predictive characteristics. The functional form of continuous characteristics (age, albumin, ALP, GGT and transaminase) was assessed by plotting each against the Martingale residuals and, subsequently, appropriate transformations were carried out where necessary. Clinically important two-way predictor interactions were also investigated and were included in the final model if they were statistically significant. The significant predictors from the Weibull model were then refitted to different parametric model distributions including the generalised gamma, log-logistic, log-normal, and exponential distributions to find the one that fitted best [19]. The Akaike's information criterion (AIC) was used to select the optimal model. Covariates which were just outside the significance level for the Weibull model were also added to these other models to check if they became significant. If they did then they were included in that model.

The problem of missing data occurs in almost all retrospective studies using routine health databases. The simplest way of dealing with this is to use only cases with complete data in the analysis. However, this leads to the loss of potentially valuable information from the incomplete cases (and hence loss of power), and introduces bias, especially if there are systematic differences between the complete and incomplete cases. Therefore, the findings from an analysis using only the complete cases may not be a true reflection on what would be found if all the cases were analysed. To analyse only the complete data assumes that the missing data are missing completely at random (MCAR) which is unlikely [20,21]. A weaker

For peer review only

version of the MCAR assumption is the missing at random (MAR) assumption. This differs from MCAR in that it assumes the missing data are dependent on one or more variables in the observed data [21]. Assuming MAR, Aa multiple imputation technique using the Markov Chain Monte Carlo (MCMC) method was conducted to impute missing values for the LFTs using PROC MI in SAS [2219]. Every model was fitted to 30 imputed datasets and the 30 sets of parameter estimates and covariances were combined to produce inferential results using PROC MIANALYZE. All baseline characteristics, time to liver disease (or censored event) and liver disease diagnosis outcome were included in the procedure. The complete data were also analysed separately as a sensitivity analysis.

The integrated discrimination index (IDI) was used to measure the improvement in the final model for each individual covariate [2023]. The IDI for a covariate is essentially the difference between the proportion of variance explained by the full model (i.e. adjusted for all covariates) and the model without the covariate of interest. The sensitivity, specificity, PPV, and negative predictive value (NPV) were also calculated for different risk cut-offs, accounting for censoring [2023].

Model assessment

The final model was assessed for its predictive ability. The C-statistic was used as a measure of discrimination [21]. Discrimination assesses the model's ability to correctly distinguish between patients who have liver disease (for whom the model assigns a high risk) and do not have liver disease (for whom the model assigns a low risk). The model's predicted probabilities were assessed for accuracy using calibration plots (further information in Appendix 2) [22]. A calibration slope test was conducted to test for overfitting of the model

For peer review only

~~[22]. This was done by fitting the linear predictor of the final model in a model by itself and testing its slope. A significant deviation from one signifies overfitting.~~

Model validation

A separate cohort of patients was obtained from the Primary Care Clinical Informatics Unit (PCCIU), University of Aberdeen [24~~3~~], and was used to externally validate the final model. The validation cohort contained all patients registered with 19 practices from across Scotland out-with Tayside. The practices were participating in the Practice Team Information project operated by the Information Services Division of the National Health Service National Services Scotland, and contributed data to PCCIU. The patient population within the PCCIU database is broadly representative of the Scottish population, with respect to age, sex, and social deprivation [25~~4~~]. The validation cohort contained patients having their initial LFTs measured in primary care between January 2004 and August 2008. All eligible patients had to have test results for ALP, bilirubin, albumin, and transaminase. All baseline characteristics and outcome data obtained for the derivation cohort were also obtained for the validation cohort. The same exclusion criteria listed above was also applied to the validation cohort.

The parameter estimates of the final model from the derivation cohort were applied to the validation cohort to ~~calculate predicted probabilities of liver disease.~~ assess its predictive ability. The C-statistic was used as a measure of discrimination [26]. Discrimination assesses the model's ability to correctly distinguish between patients who develop liver disease (for whom the model assigns a high risk) and do not develop liver disease (for whom the model assigns a low risk). The model's predicted probabilities were assessed for accuracy using calibration plots [27]. A calibration slope test was conducted to test for over-fitting of the model and true differences in the effects of predictors [27]. This was done by fitting the

For peer review only

linear predictor of the final model in a model by itself and testing its slope. A significant deviation from one signifies over-fitting. The sensitivity, specificity, PPV, and NPV of the model were also calculated for the validation cohort. These assessments were calculated using the validation cohort with the average GGT values across the 30 imputed datasets used for those patients who did not have a GGT measurement.

~~The same model assessment methods of discrimination and calibration as detailed above were then performed.~~

Decision curve analysis

Decision curve analysis was used to determine a range of threshold predicted probabilities of liver disease where the primary care decision to refer a patient to secondary care would be better than assuming all patients are disease free (i.e. not referring anyone) and assuming that all patients have liver disease (i.e. referring everyone) [28]. The method involves plotting the net benefit of the model against the threshold probability. The net benefit is defined as the difference between the proportion of patients who are true positive and false positive weighted by the relative harm of a false positive and false negative result. The net benefit of the model is then compared to the scenario where everyone is assumed disease free and therefore not referred (net benefit equals zero) and to the scenario where everyone is assumed disease positive and therefore referred. Threshold probabilities that have a higher net benefit than both of these scenarios means that, for these thresholds, the model is better than referring no one and referring everyone. The analysis was conducted using the validation cohort and the predicted probabilities at the maximum follow-up time of 2 years were used.

Clinical scoring tool

For peer review only

A points-based scoring tool was created from a simpler version of the final model for potential use in primary care [29~~5~~]. Continuous predictors from the model were dichotomised into clinically and statistically sensible categories to create the tool. Mid-points of the categories were used to estimate the risk of liver disease for each category. The tool estimated short term risk at six months follow-up and longer term risk at two years follow-up.

Model development and validation ~~Analyses~~ were performed using SAS (v9.3) (SAS Institute, Cary, North Carolina) and the decision curve analysis was performed using the stdca() function in R version 3.0.1 [30-32].

Results

Baseline characteristics

Before applying the exclusion criteria LFTs were extracted for 310 511 patients. After excluding patients under 16 years old, non-Tayside residents and those who initial LFTs were measured in secondary care, 99 165 patients remained. After excluding those with clinically recognised liver disease at baseline the derivation cohort contained 95 977 patients with incident initial LFTs taken in primary care and with no obvious liver disease. There were more females (57.9%) than males (42.1%), and the median (interquartile range) age was 54.6 (39.2 – 68.8) years (Table 1). The most frequent co-morbidity was IHD (5.6%), followed by cancer (3.8%).

Only 8 388 (8.7%) patients had all five LFTs. The percentage of complete data for each LFT was as follows: ALP (99.2%), albumin (99.2%), bilirubin (93.6%), transaminases (76.5%), and GGT (10.9%). There were more males with complete data (i.e. having all five LFTs) than females (54.6% versus 45.4%) compared to the incomplete data group (40.9% versus 59.1%).

For peer review only

The group with complete data were also more deprived and contained more alcohol dependent patients than the incomplete data group (see Appendix 2). When those without GGT measurements were compared to those with GGT measurements the results were similar to the above since those without GGT measurements comprised the majority of the incomplete data group. The group of patients without transaminase measurements contained a higher proportion of females (63.5% versus 36.5%) than that with transaminase measurements (56.2% versus 43.8%) and had a higher median ALP result (86 U/L versus 73 U/L).

Liver disease diagnosis

A total of 481 patients (0.5%) were diagnosed with a liver disease during the two year follow-up period. Of these, 339 (70.5%) had at least one abnormal LFT (out of 22 673). Cirrhosis was the most frequently diagnosed condition (n=75, 15.6%), and in 19 of these the cause was recorded as alcohol related liver disease. PBC was diagnosed in 73 patients but only as definite in 13 (probable, n=37; possible n=23). The next most frequent diagnoses were Hepatitis C (n=68), alcoholic related liver disease (n=68), Hepatitis B (n=40), hepatocellular carcinoma (n=34), autoimmune hepatitis (n=29) and fatty liver disease (n=28).

Prediction of a liver disease

The log-normal regression model had the lowest AIC and so was proven to have the best fit to the survival distribution. The following baseline characteristics were significant predictors of liver disease diagnosis: increasing GGT, decreasing albumin, alcohol dependency, being female, increasing ALP, living in a deprived area and younger age (Table 2). There were also significant interactions between albumin and transaminase, and albumin and methadone use. NSAID use, antibiotic use, drug dependency, and all comorbidity history indicators

For peer review only

were not significantly predictive of a liver disease. The IDI statistic showed that albumin explained the greatest percentage of variance in the model followed by GGT. When the model was fitted to the subgroup of patients with complete data (N=8738), only GGT and albumin were significant out of all the predictors. However, the parameter estimates had the same direction of effect and reasonably similar size of effect. The added heterogeneity from the subgroup of patients with imputed GGT and the increased power from the much larger study population would explain the significance of all the predictors in the imputed model.

The discriminatory ability of the model was good with a C-statistic of 0.78 (95% CI 0.73, 0.83). For the model without LFTs the C-statistic was 0.59 (95% CI 0.52, 0.65), and proves that LFTs add considerable discriminatory ability to the model. The calibration curve (Appendix 2) shows reasonable agreement between the predicted and actual probabilities. However, the calibration slope deviated significantly from 1 (slope = 1.129, 95% CI 1.069 to 1.189) suggesting some evidence of overfitting. However, with large sample sizes the chance of tests being statistically significant increase and the magnitude of the difference of the slope from 1 is not particularly large.

The sensitivity, specificity, PPV and NPV for different cut-offs of predicted risk of liver disease diagnosis along with the receiver operating characteristic curves for the final model applied to the derivation and external validation cohorts are displayed in Appendix 3. A cut-off greater than or equal to the 75th percentile, 0.43%, had sensitivity and specificity of approximately 75% each. The NPV was very high at almost 100% for all cut-offs which is useful when trying to avoid unnecessary tests. The PPV was low since liver disease diagnosis was relatively rare. These performance measures were similar for both derivation and validation cohorts.

For peer review only

Validation

After exclusions, the external cohort contained 11 653 patients with incident initial LFTs taken in primary care. A total of 57 patients (0.5%) were diagnosed with a liver disease within two years. The proportion of males and females were reasonably similar to the derivation cohort (45.2% versus 42.1% males), but the external cohort was older, more deprived and had a greater proportion of co-morbidities and medications (Table 1). However, the median LFTs were similar between the two cohorts. GGT was missing for 4178 (35.9%) patients and was imputed using the same method as for the derivation cohort. The average GGT measure for each patient from the 30 imputed datasets was used where the original measure was missing so that the validation could be performed in one dataset. ~~A total of 57 patients (0.5%) were diagnosed with a liver disease within two years.~~

The C-statistic for the final model parameter estimates applied to the external cohort was 0.78 (95% CI 0.72 to 0.84). The calibration plot (Appendix 3~~2~~) showed some visible evidence of over-fitting for the highest risk group, however the calibration slope showed no significant deviation from 1 (slope = 0.871 (95% CI 0.696 to 1.047); p=0.15).

The sensitivity, specificity, PPV and NPV for different cut-offs of predicted risk of liver disease diagnosis along with the receiver operating characteristic curves for the final model applied to the derivation and external validation cohorts are displayed in Appendix 4. A cut-off greater than or equal to the 75th percentile of the risk score (i.e. 0.43% in the derivation cohort and 0.57% in the validation cohort) had sensitivity and specificity of approximately 75% each. The NPV was very high at almost 100% for all cut-offs. Although PPV was very low it rose to over 10% for predicted risks over 5% and reached 20-30% for higher risks

For peer review only

(over 20%). These performance measures were similar for both derivation and validation cohorts. From the decision curve in Figure 1 it can be seen that the range of threshold predicted probabilities where the prediction model is of value is approximately between 0.5% and 7.5%.

Predicted probabilities

The final model and how to calculate the risk of liver disease directly from it is presented in Appendix 54. Figure 21 shows the risk of diagnosis over two years for the average risk patient and two example patients with different characteristics as described in the legend.

Clinical scoring tool

A user friendly paper-based scoring tool was developed from a slightly simpler version of the final model with the albumin and transaminase interaction term removed (Figure 32). The C-statistic for the scoring tool when applied to the validation cohort was 0.77 (95% CI 0.59 to 0.89). When the tool's approximation to the linear predictor was used in a calibration slope test it was not significantly different from one, Beta=0.93 (0.73 to 1.14), p=0.53. The scores for each of the risk factors add up to a total score that corresponds to the risk of liver disease. The baseline case for the tool was a 45 year old female, living in an affluent area, who was not alcohol or methadone dependent with transaminase=11.5U/L, ALP=46 U/L, GGT=17 U/L and albumin=48g/L. Her score of zero corresponded to a liver disease risk of 0.03%. As an example, a 60 year old male living in a deprived area who is alcohol dependent, but not a methadone user, with transaminase=70U/L, ALP=130U/L, GGT=45U/L and albumin=38 has a score of 29 which corresponds to a liver disease risk within two years of 4.1%. For

For peer review only

comparison, when this patient's characteristics were entered into the full model in appendix 54 their risk was 3.8%.

The correlation between the predicted probabilities from the scoring tool probabilities and the predicted probabilities from the final model when applied to the validation cohort was 0.91. Appendix 6 shows the scatterplot of the relationship between the two.

Discussion

Summary

A user-friendly clinical scoring tool has been developed that can estimate the risk of liver disease diagnosis at six months and two years in patients who had their initial LFTs measured in primary care. The tool is based on a clinical prediction model which was derived and validated using large population databases. This model adds morbidity and a longer duration to a previous algorithm that predicts one year mortality as part of our Algorithms for Liver Function Investigations (ALFI) [3326].

Strengths and limitations

This is the first large-scale population-based analysis of LFTs with complete determination of liver disease outcome. ALFI has been derived from unselected "real-world" observations in a geographically defined population: an approach recommended by the National Institutes of Health [3427]. A weakness of the electronic databases was the lack of some potential predictors of liver disease e.g. alcohol intake and body mass index, which the GP can readily ascertain to guide decisions. However, predictors in the model such as LFTs and deprivation may act as surrogate markers for such factors. Whilst we have no data on the clinical indications for LFTs being measured, we were able to identify patients with a history of

For peer review only

major co-morbidities since 1980, including cancer, diabetes and IHD using hospital admission records and population registers, and patients who were prescribed statins, NSAIDs, and antibiotics. Using the predictors we did have, the model was proven to have good ability to predict liver disease. There will have been underascertainment of some liver diseases; particularly those diagnosed in primary care, such as mild alcohol related liver disease and the steatotic end of the spectrum of non-alcoholic fatty liver disease. Patients with these diseases are most likely to be dealt with by being advised to lose weight or reduce alcohol intake, and only those who developed further problems and who were referred would be captured on the database.

The aim of the study was to combine known significant predictors of liver disease into one source of prognostic information that could provide the GP with an individualised estimate of the predicted risk of liver disease at the point where the patient has had their initial LFTs measured. If, at that point, the patient has a high risk of liver disease based on this model then the GP can also use other contextual information and their own clinical judgement to make a decision as to whether to monitor or refer the patient to a liver clinic according to national or local protocols.

The predicted probabilities from the scoring tool correlated well with those from the model. However, it is evident from the scatterplot in Appendix 6 that for high predicted probabilities from the final model the tool under-estimates the predicted probability. This is because many of these higher probabilities come from patients who have very highly abnormal LFTs. The tool assigns such patients the points based on the highest cut-off in the tool. For example, if a patient has an ALP measure of 825 the tool assigns the same points as someone with an ALP of 181 (i.e. +4 points) since >=180U/L is the highest cut-off in the tool. It would be

For peer review only

inefficient to expand the number of groups for the LFTs to include such large values as the score card would be huge. In such circumstances the GP would automatically refer without the need for a scoring tool. Also, it is advisable to reduce the influence of extreme values when creating such a scoring tool by using the 99th percentile value of measurements as the largest possible value when creating the cut-offs [29].

External validation

The validation cohort came from 19 general practices across nine different regions of Scotland during a different time period than the derivation cohort. Good discrimination and calibration demonstrated that ALFI is transportable to different geographical and temporal populations. The C-statistic for discriminatory ability was 0.78 which is comparable to that found in the validation of the Framingham equation on different cohorts ranging from 0.63 to 0.83 [3528]. The external cohort also contained more deprived and comorbid patients than the derivation cohort proving that ALFI is also robust to changes in baseline characteristics.

GGT and transaminase

GGT and, to a lesser extent, transaminase were either missing or not tested for a large proportion of patients in the derivation cohort but much less so in the validation cohort. From the database, it was not possible to determine the exact reason for the non-presence of these two LFTs. However, in Tayside, the laboratories do not routinely include GGT with the other four LFT results unless specifically requested by the primary care physician. The demographics of the patients with complete data (i.e. males, illicit drug users, alcohol dependents, and patients living in deprived areas) suggested that some primary care physicians may have requested GGT where they suspected substance abuse.[7] Testing bone biochemistry may explain the reason for transaminase not being measured since this group

For peer review only

contained a higher proportion of females who are more susceptible to bone disease, such as osteoporosis, and had a higher median ALP which is a marker for bone disease. Furthermore, one of the hospitals in Tayside analysed transaminase using a separate analyser for several years throughout the study and did not keep electronic copies which may also explain some of the missing values. Therefore it was assumed that the missing/untested LFT data depended on variables in the observed data, the assumption required for multiple imputation, [22] and the appropriate guidelines for handling this problem were followed [36].

Implications for practice and research

At a cut-off of 0.6~~43~~% (the 75th percentile) for liver disease risk, sensitivity and specificity were similar with values of ~~74.1% and 75.2% respectively~~73.7% and 75.3% in the validation cohort. However, the PPV for ~~this~~ low cut-offs such as this was poor ~~at 1.5%~~. The specificity at a cut-off of 1.2% risk is greater than 90% and rises to 100% at the 10% cut-off. ~~and~~ The NPV of the model are very high even for reasonably small cut-offs of risk meaning that the model is good at ruling out risk of liver disease within two years. However, this is not surprising given the low prevalence of liver disease in the sample of 0.5% [~~29~~37]. The PPV was low since liver disease diagnosis was relatively rare but it rose to over 10% for predicted risks of over 5% and in a small minority of patients with higher predicted risk reached 20-30% with the NPV remaining very high. ~~For example, a cut-off of 5% (specificity 98.9% and NPV 99.6%) could be used to rule out patients with lower risk in combination with clinical judgement. Such an approach could avoid unnecessary further investigations.~~ Therefore ALFI may be most accurate at higher predicted risk cut-offs with reasonable PPVs. As well as displaying reasonable accuracy, it is also important to show that the model has the potential to improve decision making.

For peer review only

The decision curve analysis showed that the model was of value for threshold probabilities between 0.5% and 7.5%. To determine whether the model is of clinical value, we should consider the possible range of threshold probabilities of liver disease at which GPs would decide to refer the patient to secondary care. The decision to refer is not a particularly risky intervention for the patient. Initially, it will lead to further blood tests and possible ultrasound in secondary care. The outcome of these results will determine whether the patient requires a liver biopsy which has some risk attached to it. However, if the patient gets to that stage then the clinician must have a high suspicion of liver disease, meaning that the decision to refer was correct. If the GP does not refer and the patient does have or develop liver disease, then the risk to the patient (i.e. of being a false negative) depends on the type and stage of the disease. Therefore, the GP may think that the intervention of referral is much less risky than not referring and decide that a probability threshold of, say, 5% is enough to refer. For thresholds between 5-7.5% the model is also reasonably accurate. Therefore ALFI could help GPs focus referral on a small subset of patients with higher predicted risk and reasonable PPVs whilst taking clinical factors that were not included in the model (but which may have improved prediction) into account. However, where the risk is lower, the long timeframe of the development of many liver diseases means that GPs can continue to address modifiable liver disease risk factors (e.g. alcohol misuse). ~~general practitioners should also counsel over liver disease risk e.g. alcohol misuse~~. The next step is to ~~quantify the clinical usefulness of ALFI and~~ evaluate ~~it~~ ALFI as a complex intervention whilst taking into account the cost associated with referral of false positives [38~~0~~].

For peer review only

For peer review only - http://bmjopen.bmj.com/site/about/guidelines.xhtml

GGT should be considered just as important as other LFTs in the prediction of liver disease since only albumin explained more variation in the model. Proper use of ALFI in practice will depend on GGT being a routine part of the LFT panel. Local health economies will have to decide if the additional cost of an extra test in the panel is worth it in terms of GGT's value in improving the ability to predict those patients who at high (or low) risk and, subsequently, change referral practice. Before such decisions are made further research is necessary involving cost-effectiveness analysis.

*

GGT was missing for a large proportion of patients in the derivation cohort but much less so in the validation cohort. Since only albumin explained more variation in the model, GGT should be considered just as important as other LFTs in the prediction of liver disease.

Conclusions

In summary, this study has developed and externally validated the ALFI model for prediction of liver disease diagnosis in patients with no clinically obvious liver disease having their LFTs taken within primary care. From this model, a simple scoring tool was developed to facilitate GP decision making with regards to retesting or referring their patients. GP decisions regarding referral of patients to secondary care should be based on probability thresholds where benefits outweigh harm [39]Its main strength is to accurately 'rule out' patients who have low risk of being diagnosed with a liver disease. This ALFI requires further evaluation as a complex intervention but it has the potential to save health service costs and prevent unnecessary further investigations and treatments.

Acknowledgements

The authors wish to thank Alison Bell from HIC for extracting and pseudonymising all the datasets used. We thank Primary Care Clinical Informatics Unit (PCCIU) at the University of Aberdeen for providing the Practice Team Initiative cohort for external validation of our model.

Funding

This work was supported by the UK National Health Service Research & Development Programme Health Technology Assessment Programme (project number 03/38/02) and supported by the Backett Weir Russell Career Development Fellowship.

Department of Health Disclaimer: The views and opinions expressed therein are those of the authors and do not necessarily reflect those of the Department of Health.

The study funder did not participate in the study design; in the collection, analysis and interpretation of data; in the writing of the report; or in the decision to submit the article for publication.

Competing interests

None declared.

Authors' contributions

DJM contributed to the design of the study, conducted the analyses, drafted and finished the paper. JFD, FMS, PR, WR and SR contributed to the design and conduct of the study, and the writing of the paper. PTD contributed to, and oversaw the design and conduct of the study, and contributed to the writing of the paper. He is the guarantor for the paper. All authors read and approved the final manuscript.

For peer review only

References

1. Pratt DS, Kaplan MM: **Evaluation of abnormal liver-enzyme results in asymptomatic patients**. *N Engl J Med* 2000, **342**:1266-1271.

2. Schalk BWM, Visser M, Bremmer MA, Penninx BWJH, Bouter LM, Deeg DJH: **Change of serum albumin and risk of cardiovascular disease and all-cause mortality: Longitudinal Aging Study Amsterdam**. *Am J Epidemiol* 2006, **164**:969-977.

3. Sorensen HT, Moller-Petersen JF, Felding P, Andreasen C, Nielsen JO: **Epidemiology of abnormal liver function tests in general practice in a defined population in Denmark**. *Dan Med Bull* 1991, **38**:420-422.

4. Kim HC, Nam CM, Jee SH, Han KH, Oh DK, Suh I: **Normal serum aminotransferase concentration and risk of mortality from liver diseases: Prospective cohort study**. *Br Med J* 2004, **328**:983-986.

5. Roderick P: **Commentary: Liver function tests: defining what's normal**. *Br Med J* 2004, **328**:987.

6. Sherwood P, Lyburn I, Brown S, Ryder S: **How are abnormal results for liver function tests dealt with in primary care? Audit of yield and impact**. *Br Med J* 2001, **322**:276-278.

7. McLernon DJ, Donnan PT, Ryder S, Roderick P, Sullivan FM, Rosenberg W, Dillon JF: **Health outcomes following liver function testing in primary care: a retrospective cohort study**. *Fam Pract* 2009, **26**:251-259.

8. Moons K, Royston P, Vergouwe Y, Grobbee D, Altman D: **Prognosis and prognostic research: what, why, and how?** *Br Med J* 2009, **338**:b375.

9. Fornasini M, Brotons C, Sellarès J, Martinez M, Galán ML, Sáenz L, da Pena JM: **Consequences of using different methods to assess cardiovascular risk in primary care**. *Fam Pract* 2006, **23**:28-33.

For peer review only

10. Siris ES, Baim S, Nattiv A: **Primary care use of FRAX: absolute fracture risk assessment in postmenopausal women and older men**. *Postgrad Med* 2010, **122**:82-90.

11. Altman DG, Vergouwe Y, Royston P, Moons KGM: **Prognosis and prognostic research: validating a prognostic model**. *Br Med J* 2009, **338**:b605.

12. Donnan PT, McLernon D, Dillon JF, Ryder S, Roderick P, Sullivan F, Rosenberg W: **Development of a decision support tool for primary care management of patients with abnormal liver function tests without clinically apparent liver disease: a record-linkage population cohort study and decision analysis (ALFIE)**. *Health Technol Assess* 2009, **13**(25).

13. Carstairs V, Morris R: **Deprivation and mortality: an alternative to social class?** *Community Med* 1989, **11**:210-219.

14. Evans JMM, MacDonald TM: **Record-linkage for pharmacovigilance in Scotland**. *Br J Clin Pharm* 1999, **47**:105-110.

15. NHS National Services Scotland: **SMR01 – General/Acute Inpatient and Day Case** [http://www.datadictionary.scot.nhs.uk/isddd/9065.html]

16. Morris AD, Boyle DI, MacAlpine R, Emslie-Smith A, Jung RT, Newton RW, MacDonald TM: **The diabetes audit and research in Tayside Scotland (darts) study: electronic record linkage to create a diabetes register**. *Br Med J* 1997, **15**:524-528.

17. Donnan PT, Wei L, Steinke DT, Phillips G, Clark R, Noone A, Sullivan FM, Macdonald TM, Davey PG: **Presence of bacteriuria caused by trimethoprium resistant bacteria in patients prescribed antibiotics: multilevel model with practice and individual patient data**. *Br Med J* 2004, **328**:1297-1301.

18. Steinke DT, Weston TL, Morris AD, MacDonald TM, Dillon JF: **The epidemiology of liver disease in Tayside database: a population-based record-linkage study**. *J Biomed Inform* 2003, **35**:186-193.

For peer review only

19. Collett D. *Modelling survival data in medical research.* 2 ed. Boca Raton: Chapman & Hall/CRC, 2003.

20. Rubin DB: **Inference and missing data**. *Biometrika* 1976, **63**:581–592.

2119. Rubin DB: *Multiple imputation for nonresponse in surveys*. New York: Wiley; 1987.

22. Yuan YC: **Multiple imputation for missing data: concepts and new development**: SAS Institute Inc. [http://support.sas.com/rnd/app/papers/multipleimputation.pdf].

230. Chambless LE, Cummiskey CP, Cui G: **Several methods to assess improvement in risk prediction models: extension to survival analysis**. *Stat Med* 2011, **30**:22-38.

21. Pencina MJ, D'Agostino RB: **Overall C as a measure of discrimination in survival analysis: model specific population value and confidence interval estimation**. *Stat Med* 2004, **23**:2109-2123.

22. Steyerberg EW: *Clinical prediction models: a practical approach to development, validation, and updating*. New York: Springer; 2009.

243. University of Aberdeen: **Primary Care Clinical Informatics Unit** [http://www.abdn.ac.uk/pcciu/index.htm]

254. Information Services Division Scotland: **General Practice – Practice Team Information (PTI)** [http://www.isdscotland.org/isd/1283.html#Background_to_PTI]

26. Pencina MJ, D'Agostino RB: **Overall C as a measure of discrimination in survival analysis: model specific population value and confidence interval estimation**. *Stat Med* 2004, **23**:2109-2123.

27. Steyerberg EW: *Clinical prediction models: a practical approach to development, validation, and updating*. New York: Springer; 2009.

28. Vickers AJ, Elkin EB: **Decision curve analysis: a novel method for evaluating prediction models**. *Med Decis Making* 2006, **26**: 565.

For peer review only

2~~9~~5. Sullivan LM, Massaro JM, D'Agostino Sr RB: **Tutorial in biostatistics: Presentation of multivariate data for clinical use: The Framingham Study risk score functions**. *Stat Med* 2004, **23**:1631-1660.

30. R Core Team. **R: A language and environment for statistical computing.** R Foundation for Statistical Computing, Vienna, Australia 2014 [http://www.R-project.org].

31. Vickers AJ, Memorial Sloan Kettering Cancer Centre: **Epidemiology and Biostatistics: Decision curve analysis**. [http://www.mskcc.org/research/epidemiology-biostatistics/health-outcomes/decision-curve-analysis-0].

32. Vickers AJ, Cronin AM, Elkin EB, Gonen M: **Extensions to decision curve analysis, a novel method for evaluating diagnostic tests, prediction models and molecular markers.** *BMC Med Inform Decis Mak*, **8**:53.

33~~26~~. McLernon DJ, Dillon JF, Sullivan FM, Roderick P, Rosenberg WM, Ryder S, Donnan PT: **The utility of liver function tests for mortality prediction within one year in primary care using the Algorithm for Liver Function Investigations (ALFI)**. *PLoS ONE* 2012, **7**:e50965.

34~~27~~. Dorans K: **Pilot projects aim to ease access to clinical data**. *Nature Med* 2009, **15**: 226.

35~~28~~. D'Agostino RB, Grundy S, Sullivan LM, Wilson P: **Validation of the Framingham Coronary Heart Disease Prediction Scores. Results of a multiple ethic groups investigation**. *JAMA* 2001, **286**:180-187.

36. Sterne JAC, White IR, Carlin JB, Spratt M, Royston P, Kenward MG, Wood AM, Carpenter JR: **Multiple imputation for missing data in epidemiological and clinical research: potential and pitfalls**. *Br Med J* 2009, **338**:b2393.

~~29~~37. Hawkins RC. **The evidence based medicine approach to diagnostic testing: practicalities and limitations**. *Clin Biochem Rev* 2005, **26**:7-18.

For peer review only

3~~8~~0. Craig P, Dieppe P, Macintyre S, Mitchie S, Nazareth I, Petticrew M. **Developing and evaluating complex interventions: the new Medical Research Council guidance**. *Br Med J* 2008, **337**:a1655.

39. Goodwin N. **Diagnostic delays and referral management schemes: how 'integrated' primary care might damage your health**. *Int J Integr Care* 2008, **8**:e78.

For peer review only

Box 1: Databases record-linked to create the Tayside derivation cohort

1. Death registry from the General Register Office for Scotland including date and causes of death.
2. Deprivation score was assessed by the Carstairs method [13]. The score was divided into two categories of affluent and deprived.
3. Tayside Community Health Index (CHI) files. Contains CHI number [14], name, address and date of birth for all individuals registered with a general practitioner in Tayside. It also holds migration dates to and from Tayside. The database was pseudonymised before release to the researcher.
4. Regional biochemistry database. All LFTs came from the largest hospital laboratory in the Tayside region, Ninewells. Two smaller hospital laboratories contributed electronic LFT results later, one from 1998 and the other from 2003.
5. Scottish Morbidity Records 1 (SMR01) database: Patient specific morbidity data are routinely collected in Scotland and collectively these are known as Scottish Morbidity Records (SMR). SMR01 contains hospital admissions and procedures for all hospitals in Scotland and is one of the oldest and most complete national health datasets in the world [15]. Major co-morbidity groups at baseline were identified using this database, and the Diabetes Audit and Research Tayside Scotland (DARTS) and Heart disease Evidence-based Audit and Research Tayside Scotland (HEARTS) databases for diabetes and chronic heart disease respectively [16].
6. SMR04 database: Holds all inpatient and day case episodes for mental health specialties, which identified patients admitted/discharged with diagnoses of alcohol dependency or drug misuse.
7. Pharmacist dispensed prescription database: A community based database that holds encashed prescriptions in Tayside [17], and allowed identification of patients on some potentially hepatotoxic drugs including statins, antibiotics, and Non-Steroidal Anti-Infammatory Drugs (NSAIDs).
8. Epidemiology of Liver Disease In Tayside (ELDIT) database: Contains all Tayside patients with liver diseases that have been 'electronically' diagnosed using record-linkage of biomedical datasets, including virology and immunology [18]. ELDIT was used for the study exclusion criteria (i.e. previous liver disease, liver disease complications within 6 weeks) and for the outcome of liver disease diagnosis during the two-year follow-up period.

Table 1 Baseline characteristics of patients in the derivation (n=95977) and validation (n=11653) cohorts

Baseline Characteristics		Cohort n(%) *or* Median (IQR)	
		Derivation	Validation
Age (years)		54.6 (39.2-68.8)	60.0 (47.0, 72.0)
Gender	Male	40374 (42.1)	5271 (45.2)
	Female	55603 (57.9)	6382 (54.8)
Carstairs category[1]	Affluent	47286 (49.3)	2753 (23.6)
	Deprived	48691 (50.7)	8900 (76.4)
Comorbidity history	Cancer[2]	3629 (3.8)	956 (8.2)
	Diabetes	1386 (1.4)	1441 (12.4)
	IHD	5370 (5.6)	2034 (17.5)
	Renal disease	141 (0.2)	155 (1.3)
	Respiratory disease	2636 (2.8)	883 (7.6)
	Stroke	1471 (1.5)	583 (5.0)
Medication in previous 3 months	Statins	3176 (3.3)	3178 (27.3)
	NSAIDs	6698 (7.0)	1762 (15.1)
	Antibiotics	8307 (8.7)	1962 (16.8)
Abusive substance	Alcohol	2632 (2.7)	465 (4.0)
	Drug	371 (0.4)	0 (0.0)
	Methadone	377 (0.4)	10 (0.1)
Liver function tests	Albumin (g/L)	44.0 (42.0-46.0)	44.0 (41.0, 46.0)
	ALP (U/L)	76.0 (62.0-94.0)	75.0 (62.0, 92.0)
	Transaminase (U/L)	18.0 (14.0-26.0)	21.0 (16.0, 30.0)
	GGT (U/L)	26.0 (17.0-47.0)	27.0 (18.0, 45.0)
	Bilirubin[3]		
	Normal	81111 (91.0)	10587 (90.8)
	Mildly raised	8058 (9.0)	1066 (9.2)

Data reported are median (interquartile range) or percentage

For peer review only

[1]Carstairs categories 1 to 3 were recoded as affluent and categories 4 to 7 were recoded as deprived.

[2]Not including biliary cancer or hepatocellular cancer;

[3]Normal bilirubin defined as 0-15□mol/L for females, 0-17μmol/L for males; Mildly raised bilirubin defined as 16-35 μ mol/L for females, 18-35 μ mol/L for males.

IQR interquartile range; IHD ischaemic heart disease; NSAID non-steroidal anti-inflammatory; ALP alkaline phosphatase; GGT gamma-glutamyltransferase.

For peer review only

Table 2 Parameter estimates (95% CI) and IDI for the final log-normal regression model predicting risk of a liver disease diagnosis within 2 years of initial liver function tests (481 diagnosed)

Parameter	Coefficient (95% CI)	P-value	IDI, %
Intercept	9.524 (1.986 to 17.062)	0.013	
Albumin	0.488 (0.306 to 0.669)	<0.001	0.711[a]
Log(GGT)	-1.704 (-2.223 to -1.184)	<0.001	0.689
Methadone (yes *v* no)	8.319 (-2.019 to 18.657)	0.115	0.465[a]
Log(ALP)	-0.739 (-1.213 to -0.264)	0.002	0.307
Log(transaminase)	2.016 (-0.151 to 4.183)	0.068	0.266[a]
Alcohol dependent (yes *v* no)	-1.210 (-1.856 to -0.563)	<0.001	0.143
Gender (Male *v* Female)	0.583 (0.236 to 0.930)	0.001	0.135
Age at baseline	0.014 (0.004 to 0.024)	0.009	0.121
Deprived[b] (yes *v* no)	-0.518 (-0.852 to -0.183)	0.002	0.013
Methadone X Albumin	-0.295 (-0.530 to -0.061)	0.014	
Albumin X Log(transaminase)	-0.070 (-0.122 to -0.018)	0.008	
Scale	4.551 (4.192 to 4.910)	<0.001	

GGT = gamma-glutamyltransferase; ALP = alkaline phosphatase; IDI=integrated discrimination improvement.

[a] the relative interaction terms containing this parameter were also excluded.

[b] Carstairs categories 1 to 3 were coded as affluent and categories 4 to 7 were coded as deprived.

Note: Parameter estimates are in decreasing order of IDI. A negative coefficient indicates an increased risk of diagnosis, whilst a positive coefficient indicates a decreased risk of diagnosis. However, this differs for terms involved in interactions i.e. increasing transaminase increases risk; for a methadone user, increasing albumin increases risk; for non-methadone users, decreasing albumin increases risk.

For peer review only

Figure titles and legends

Figure 1. Decision curve for a model to predict liver disease diagnosis in patients having their LFTs measured in primary care.

Dashed line: Prediction model; grey line: assume all patients have liver disease; black line: assume no patients have liver disease.

Figure 21. Probability of a liver disease diagnosis during the two years for the average risk patient and two example patients with different risk levels.

Note: Average risk is defined as the risk calculated using the average linear predictor value from the model. Patient 1 and Patient 2 characteristics are presented in the boxes within the plot. The estimated risk of being diagnosed with a liver disease within two years for each of these patients is 0.002 (average risk patient), 0.097 (Patient 1) and 0.493 (Patient 2).

Figure 32. Clinical scoring tool for likely liver disease diagnosis in primary care.

For each risk factor category enter the corresponding score into the box on the right hand side. Sum the scores in the total score box. Look for the total score in the lower table and read off the risk of liver disease within 6 months and/or 2 years.

For peer review only

List of Supplementary Materials

File name: Appendix 1

File format: .docx

Title of data: Liver diseases from the ELDIT database

Description of data: Liver diseases from the ELDIT database used as the outcome in the model and comorbidities used as potential predictors of the outcome along with their original clinical database source(s)

File name: Appendix 2

File format: .doc

Title of data: Complete case analyses

Description of data: Comparison of baseline characteristics in patients with complete data for all five LFTs with patients with incomplete data. Model fitted to complete data only.

File name: Appendix 32

File format: .docx

Title of data: Calibration plots

Description of data: Calibration plots for the final model applied to the development cohort and to the validation cohort

File name: Appendix 43

File format: .docx

Title of data: Performance of the final model to predict liver disease diagnosis

Description of data: Receiver operating characteristic (ROC) curves for the final model to predict liver disease diagnosis applied to the derivation cohort and the external validation

For peer review only

cohort. Sensitivity, specificity, positive predictive value (PPV), and negative predictive value (NPV) for different percentiles of liver disease risk for the derivation and validation cohorts are also presented.

File name: Appendix 54

File format: .docx

Title of data: Calculating the risk of liver disease diagnosis within two years from the final model.

Description of data: A mathematical formula showing how to calculate the predicted risk of liver disease diagnosis at any time point during a two year maximum follow-up period. A worked example is also presented.

File name: Appendix 6

File format: .docx

Title of data: Scatterplot of the predicted probability of liver disease diagnosis from the full model against the predicted probability from the scoring tool.

Description of data: Scatterplot to show the correlation between the predicted probability of liver disease diagnosis from the full model and the predicted probability of liver disease diagnosis from the scoring tool.

For peer review only

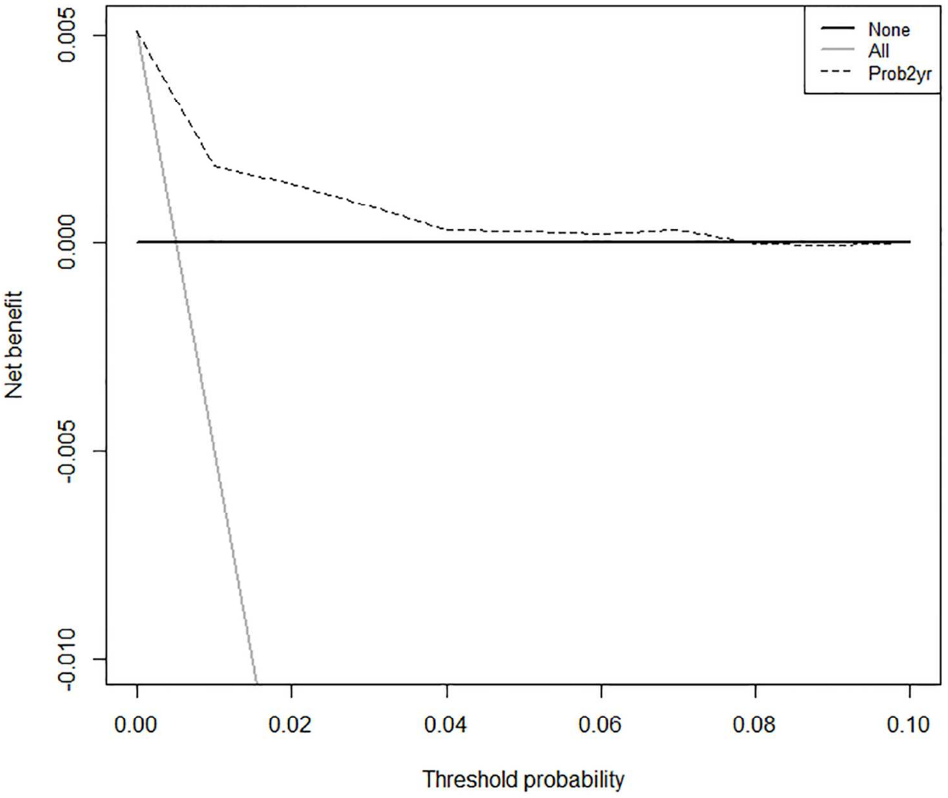

120x111mm (300 x 300 DPI)

only

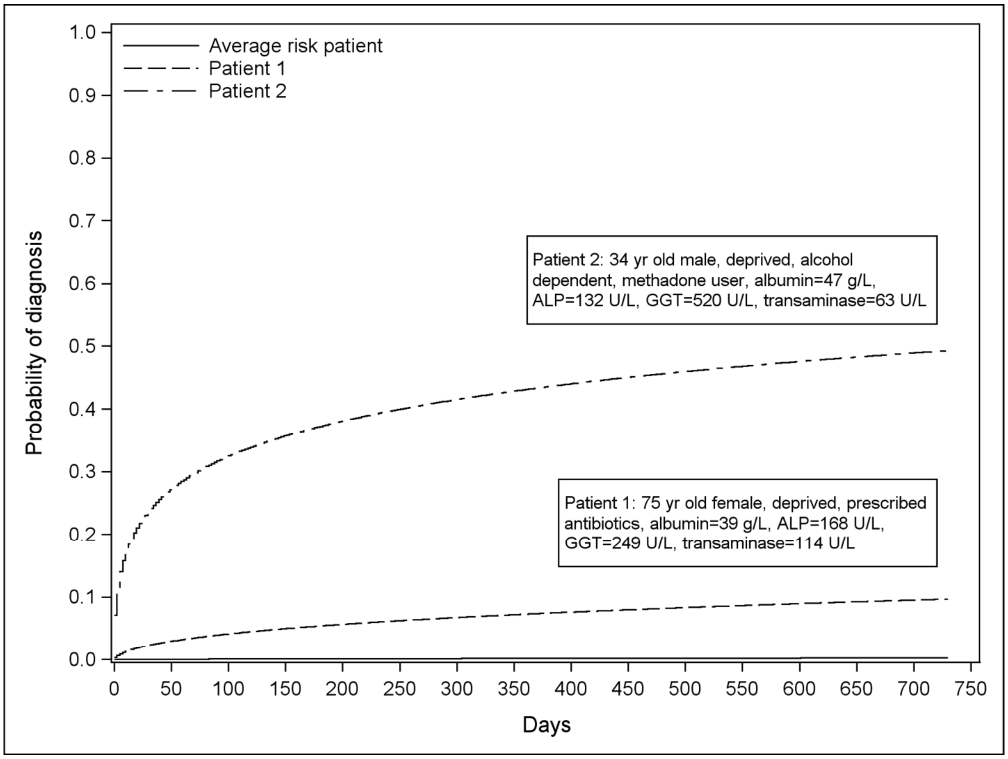

508x381mm (300 x 300 DPI)

Risk factors	Categories and corresponding score						Score
Age (Years)	16 to 35	36 to 55	56 to 75	≥75			
	+1	0	-1	-2			
Transaminase (U/L)	<20	20 to 39	40 to 59	60 to 79	≥80		
	0	+3	+5	+6	+7		
ALP (U/L)	<60	60 to 89	90 to 119	120 to 149	150 to 179	≥180	
	0	+1	+2	+3	+3	+4	
GGT (U/L)	<30	30 to 59	60 to 89	90 to 119	120 to 149	≥150	
	0	+6	+9	+12	+13	+14	
Albumin (g/L) AND	<31	31 to 35	36 to 40	41 to 45	≥46		
Methadone user	+19	+20	+21	+21	+22		
OR							Or
Not methadone user	+19	+15	+10	+5	0		
Gender	Male	Female					
	-2	0					
Deprived	No	Yes					
	0	+2					
Alcohol dependent	No	Yes					
	0	+5					
						Total score	

Estimated probability of liver disease diagnosis at 6 months and 2 years

Total Score	Liver disease probability (%)		Total Score	Liver disease probability (%)	
	within 6 months	within 2 years		within 6 months	within 2 years
5 or less	<0.1	<0.1	26	1.3	2.7
6	<0.1	0.1	27	1.5	3.1
7	<0.1	0.1	28	1.8	3.6
8	0.1	0.1	29	2.0	4.1
9	0.1	0.2	30	2.3	4.6
10	0.1	0.2	31	2.7	5.2
11	0.1	0.3	32	3.1	5.9
12	0.1	0.3	33	3.5	6.6
13	0.1	0.4	34	4.0	7.4
14	0.2	0.4	35	4.5	8.2
15	0.2	0.5	36	5.1	9.2
16	0.2	0.6	37	5.7	10.2
17	0.3	0.7	38	6.4	11.2
18	0.4	0.8	39	7.2	12.4
19	0.4	1.0	40	8.0	13.6
20	0.5	1.2	41	9.0	15.0
21	0.6	1.3	42	10.0	16.4
22	0.7	1.6	43	11.0	17.9
23	0.8	1.8	44	12.2	19.5
24	1.0	2.1	45 or more	>13.3	>21.0
25	1.1	2.4			

203x259mm (300 x 300 DPI)

Appendix 1 Liver diseases from the ELDIT database used as the outcome and comorbidities used as potential predictors of the outcome along with their original clinical database source(s)

Disease	Hospital admission diagnosis codes from SMR01 database		Other source databases from Ninewell's hospital
	ICD 9	ICD 10	
**Liver disease diagnosis**			
Hepatitis B			Virology
Hepatitis C			Virology
Autoimmune hepatitis	571.4	K73.0, K73.2, K73.8, K73.9	+ve Pathology, immunology (+ve ASM)
Cirrhosis	571.2, 571.5, 571.6	K70.3, K74.3-K74.6, K76.1	Pathology
Primary biliary cirrhosis:			
Definite			+ve Pathology, immunology (+ve AMA), biochemistry (↑GGT, ↑Alk Phos)
Probable			Any two positive tests from the above three.
Possible			Immunology (+ve AMA)
Alcoholic cirrhosis	As cirrhosis + 291, 303, 305.0	As cirrhosis + F10	SMR1/SMR4 ICD codes for alcohol
Alcoholic hepatitis	571.1	K70.1	Pathology
Alcoholic related liver disease	Any liver disease codes+alcohol codes	Any liver disease codes+alcohol codes	Pathology
Fatty liver disease	571.0, 571.8	K70.0, K76.0	Pathology
Hepatocellular carcinoma	155.0, 155.2	C22.0, C22.2-C22.9	Pathology (ICD from SMR1/SMR6)
Wilson's disease	275.1	E83.0	
Haemochromatosis	275.0, 285.0	D64.2, E83.1	Pathology
Alpha 1-antitrypsin			Biochemistry (+ve Alpha 1-antitrypsin)
**Complications of liver disease**			
Oesophageal Varices	456.1, 456.2	I85.9, I98.2A	Endoscopy records
- bleeding	456.0	I85.0	
Gastric varices		I86.4	
Ascites	789.5	R18X	
Encephalopathy	572.2	K72.9	
Portal hypertension	Any complication above or 572.3	Any complication or K76.6	
**Other liver conditions**			
Acute/subacute necrosis	570.9		
Alcoholic liver damage, unspecified	571.3	K70.9	
Chronic hepatitis	571.4		
Non-alcoholic chronic liver disease, unspecified	571.9		
Abscess of liver	572.0	K75.0	

For peer review only

Hepatorenal syndrome	572.4	K76.7	
Other sequelae of chronic liver disease	572.8		
Other liver disorder eg hepatoptosis	573.8	K76.8	
Unspec liver disorder	573.9	K76.9	
Alcoholic hepatic failure		K70.4	
Acute/subacute hepatic failure		K72.0	
Chronic hepatic failure		K72.1	
Occlusion of vena cava	453.2	I82.2	
Portal vein thrombosis	452	I81	
Hepatitis B	070	B16, B18.0, B18.1	
Hepatitis C	070	B17.1, B18.2	
Other viral hepatitis	070	B17-19	
**Comorbidities**			
Ischaemic heart disease	410-414	I20-I25	
Other cancers	140-208, 230-234 (exclude cholangiocarcinoma and hepatocellular codes)	C00-D09 (exclude cholangiocarcinoma and hepatocellular codes)	
Diabetes	250	E10-E14	
Respiratory	466, 480-496	J10-J18, J20, J40X-J47X, J66-J67	
Renal	584-586	N17-N19X, I12.0	
Stroke	430-438	I60-	

ICD=International Classification of Diseases; ASM=anti-smooth muscle antibody; AMA=antimitochondrial antibody; GGT; Alk phos=alkaline phosphatase;

Note: All diseases are definite diagnoses apart from Primary Biliary Cirrhosis which was coded as either definite, probable or possible.

For peer review only

Appendix 2: Complete case analyses

Table A1 Comparison of baseline characteristics in: patients who had all five LFTs measured with patients who did not; patients who had GGT measured with patients who did not; and patients who had transaminase measured with patients who did not

Baseline Characteristics		All LFTs		GGT		Transaminase	
		Measured (n=8388)	Missing/not measured (n=87589)	Measured (n=10484)	Missing/not measured (n=85493)	Measured (n=73360)	Missing/not measured (n=22617)
Age (years)		52.0 (39.3-65.1)	55.0 (39.2-69.1)	50.5 (38.1-63.5)	55.3 (39.4-69.3)	53.6 (38.4-68.2)	57.7 (42.2-70.2)
Gender	Male	4578 (54.6)	35796 (40.9)	5997 (57.2)	34377 (40.2)	32125 (43.8)	8249 (36.5)
	Female	3810 (45.4)	51793 (59.1)	4487 (42.8)	51116 (59.8)	41235 (56.2)	14368 (63.5)
Carstairs category	Affluent	3316 (39.5)	43970 (50.2)	4061 (38.7)	43225 (50.6)	37638 (51.3)	9648 (42.7)
	Deprived	5072 (60.5)	43619 (49.8)	6423 (61.3)	42268 (49.4)	35722 (48.7)	12969 (57.3)
Comorbidity	Cancer[1]	316 (3.8)	3313 (3.8)	365 (3.5)	3264 (3.8)	2880 (3.9)	749 (3.3)
	Diabetes	78 (0.9)	1308 (1.5)	90 (0.9)	1296 (1.5)	1164 (1.6)	222 (1.0)
	IHD	381 (4.5)	4989 (5.7)	440 (4.2)	4930 (5.8)	4238 (5.8)	1132 (5.0)
	Renal disease	10 (0.1)	131 (0.2)	10 (0.1)	131 (0.2)	105 (0.1)	36 (0.2)
	Respiratory disease	198 (2.4)	2438 (2.8)	252 (2.4)	2384 (2.8)	2021 (2.8)	615 (2.7)
	Stroke	107 (1.3)	1364 (1.6)	124 (1.2)	1347 (1.6)	1148 (1.6)	323 (1.4)
Medication in previous 3 months	Statins	182 (2.2)	2994 (3.4)	196 (1.9)	2980 (3.5)	3096 (4.2)	80 (0.4)
	NSAIDs	415 (5.0)	6283 (7.2)	563 (5.4)	6135 (7.2)	3444 (4.7)	3254 (14.4)

For peer review only

Baseline Characteristics		All LFTs		GGT		Transaminase	
		Measured (n=8388)	Missing/not measured (n=87589)	Measured (n=10484)	Missing/not measured (n=85493)	Measured (n=73360)	Missing/not measured (n=22617)
	Antibiotics	755 (9.0)	7552 (8.6)	883 (8.4)	7424 (8.7)	6218 (8.5)	2089 (9.2)
Abusive substance	Alcohol	534 (6.4)	2098 (2.4)	744 (7.1)	1888 (2.2)	1989 (2.7)	643 (2.8)
	Drug	49 (0.6)	322 (0.4)	78 (0.7)	293 (0.3)	278 (0.4)	93 (0.4)
	Methadone	43 (0.5)	334 (0.4)	58 (0.6)	319 (0.4)	307 (0.4)	70 (0.3)
Liver function tests	Albumin (g/L)	44.0 (41.0-46.0)	44.0 (42.0-46.0)	44.0 (42.0-46.0)	44.0 (42.0-46.0)	43.0 (41.0-45.0)	44.0 (42.0-46.0)
	ALP (U/L)	75.5 (61.0-95.0)	76.0 (62.0-94.0)	77.0 (62.0-97.0)	76.0 (62.0-94.0)	73.0 (60.0-90.0)	86.0 (69.0-106.0)
	Transaminase (U/L)	20.0 (15.0-30.0)	18.0 (14.0-25.0)	20.0 (15.0-29.0)	18.0 (14.0-25.0)	18.0 (14.0-26.0)	-
	GGT (U/L)	26.0 (17.0-48.0)	26.0 (17.0-46.0)	26.0 (17.0-47.0)	-	26.0 (17.0-47.0)	27.0 (16.0-49.0)
	Bilirubin[2]						
	Normal	7447 (88.8)	73664 (91.2)	8400 (89.0)	72711 (91.2)	62219 (90.3)	18892 (93.1)
	Mildly raised	941 (11.2)	7117 (8.8)	1041 (11.0)	7017 (8.8)	6667 (9.7)	1391 (6.9)
Follow-up time		731 (586-731)	731 (481-731)	731 (635-731)	731 (475-731)	731 (350-731)	731 (731-731)
Liver disease diagnosed within 2 years		77 (0.9)	261 (0.3)	95 (0.9)	243 (0.3)	267 (0.4)	71 (0.3)

Data reported are median (interquartile range) or percentage; IHD ischaemic heart disease; NSAID non-steroidal anti-inflammatory; ALP alkaline phosphatase; GGT gamma-glutamyl transferase; [1]Not including biliary cancer or hepatocellular cancer;

[2]Normal bilirubin is defined as 0-15µmol/L for females and 0-17µmol/L for males; Mildly raised bilirubin is defined as 16-35µmol/L for females and 18-35µmol/L for males.

For peer review only

The final model was fitted to the subgroup of patients with complete data to compare with the imputed data results. The complete dataset contained 8738 patients who had the four LFTs included in the final model (bilirubin was excluded since it was not statistically significant). Of these, 106 were diagnosed with a liver disease during the two year period. The only significant baseline characteristics in this model were GGT and albumin (see eTable 2). The parameter estimates of this model were not too dissimilar to those of the model derived from the imputed dataset.

Table A2 Model predicting risk of liver disease diagnosis within 2 years of initial liver function tests for patients with complete data only (N=8738)

Parameter	Coefficient (95% CI)	P-value
Intercept	3.047 (-11.754 to 17.848)	0.69
Log (GGT)	-1.209 (-1.717 to -0.700)	<0.001
Albumin	0.603 (0.244 to 0.963)	0.001
Alcohol dependent (yes vs. no)	-0.955 (-2.067 to 0.157)	0.09
Gender (Male vs. Female)	0.305 (-0.472 to 1.081)	0.44
Log (ALP)	-0.725 (-1.571to 0.120)	0.09
Deprived (yes vs. no)	-0.486 (-1.282 to 0.310)	0.23
Albumin X Log (transaminase)	-0.085 (-0.182 to 0.013)	0.09
Age at baseline	0.006 (-0.020 to -0.031)	0.67
Methadone user X Albumin	-0.347 (-0.882 to 0.188)	0.20
Log (transaminase)	2.342 (-1.685 to 6.368)	0.25
Methadone user (yes vs. no)	8.912 (-14.998 to 32.822)	0.47
Scale	4.336 (3.687 to 5.100)	

IHD ischaemic heart disease; GGT = gamma-glutamyl transferase; ALP = alkaline phosphatase

For peer review only

Appendix 4 Performance of the final model to predict liver disease diagnosis

Figure A2 Receiver operating characteristic (ROC) curve for the ALFI model to predict liver disease diagnosis in the validation cohort.

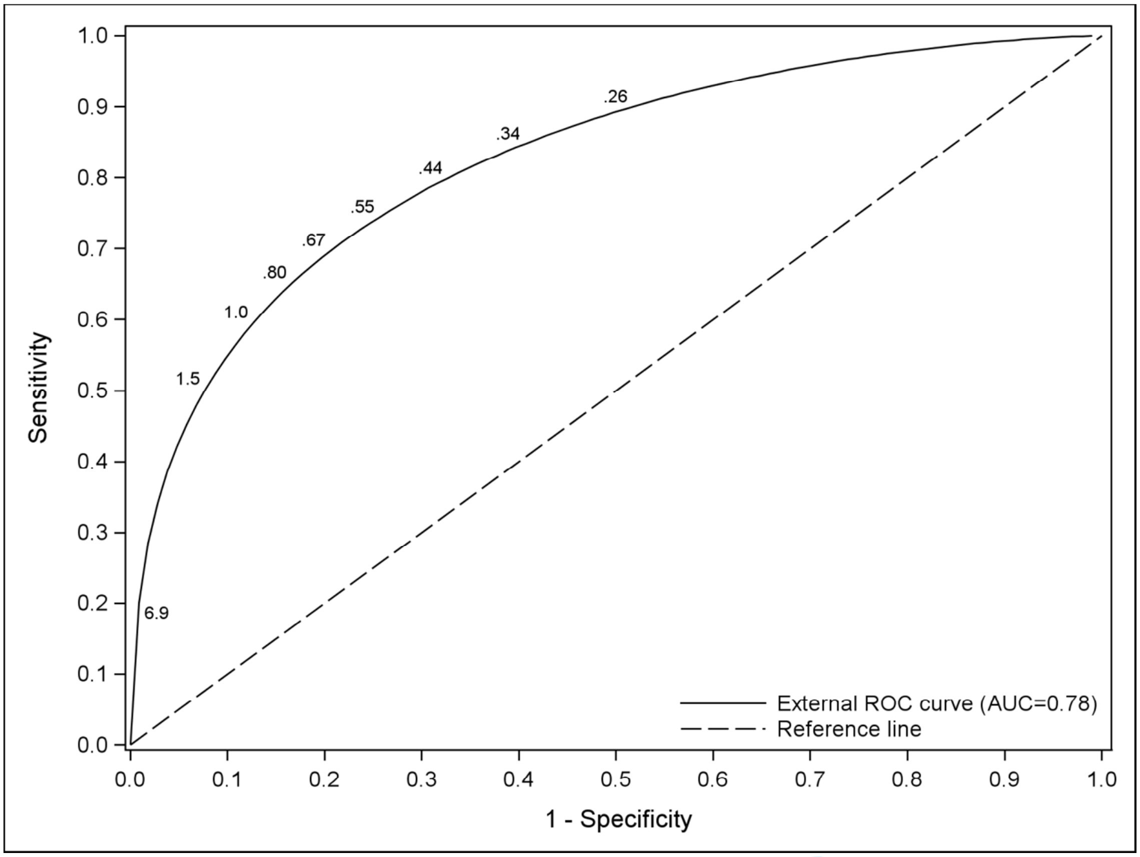

Note: The predicted risks of liver disease are displayed alongside the curve so that their sensitivity and specificity can be read.

For peer review only - http://bmjopen.bmj.com/site/about/guidelines.xhtml

Table A3 Final model sensitivity, specificity, positive predictive value (PPV), and negative predictive value (NPV) for different percentiles of liver disease risk for the derivation and validation cohorts.

Derivation cohort						Validation cohort					
Cut-off for predicted risk,%	Number (%) of patients over cut-off (out of 95,977)	Sens, %	Spec, %	PPV, %	NPV, %	Cut-off for predicted risk,%	Number (%) of patients over cut-off (out of 11653)	Sens, %	Spec, %	PPV, %	NPV, %
≥0.215[a]	47989 (50)	89.0	50.2	0.903	99.8	≥0.269 [a]	5827 (50)	89.2	50.2	1.14	99.9
≥0.427[b]	23995 (25)	74.1	75.2	1.50	99.8	≥0.569 [b]	2914 (25)	73.7	75.3	1.88	99.8
≥0.881[c]	9598 (10)	56.5	90.2	2.87	99.8	≥1.22 [c]	1167 (10)	54.5	90.3	3.46	99.7
≥1.52[d]	4799 (5)	45.3	95.2	4.59	99.7	≥2.08 [d]	583 (5)	42.1	95.2	5.35	99.6
≥5	1155 (1.20)	26.3	98.9	11.1	99.6	≥5	185 (1.59)	31.2	99.4	8.14	99.5
≥5.82[e]	960 (1.00)	24.2	99.1	12.2	99.6	≥6.95 [e]	117 (1.00)	20.1	99.1	12.6	99.5
≥10	463 (0.48)	16.5	99.6	17.3	99.6	≥10	54 (0.46)	19.2	100.0	13.1	99.4
≥20	110 (0.11)	6.53	99.9	28.7	99.5	≥20	12 (0.10)	8.03	100.0	23.8	99.4
≥30	38 (0.04)	2.93	100	36.5	99.5	≥30	5 (0.04)	4.54	100.0	30.6	99.4
≥40	10 (0.01)	1.00	100	44.4	99.5	≥40	2 (0.02)	2.47	100.0	36.6	99.4

Cut-offs for: [a]50th; [b]75th; [c]90th; [d]95th; [e]99th percentile.

For peer review only

Appendix 3: Calibration plots

In order to draw a calibration plot we have to first of all perform some calculations. First of all divide the cohort into intervals (each with 100 patients) of increasing risk of liver disease and calculating the mean *predicted* risk of liver disease at two years for each interval. Secondly, calculate the mean *actual* risk of liver disease using the Kaplan-Meier method for each interval. The mean predicted risks were then plotted against the mean actual risks. A model with perfect calibration would see the points lie along the diagonal.

Figure A1 shows reasonable agreement between the predicted and actual risks for the model in the validation cohort. The calibration plot for the model applied to the external validation cohort (Figure A1) showed some visible evidence of over-fitting for the highest risk group, however the calibration slope showed no significant deviation from 1 (slope = 0.871 (95% CI 0.696 to 1.047); p=0.15).

Figure A1: Calibration plot showing the relationship between the predicted and actual risks of survival for the final model applied to the external validation cohort

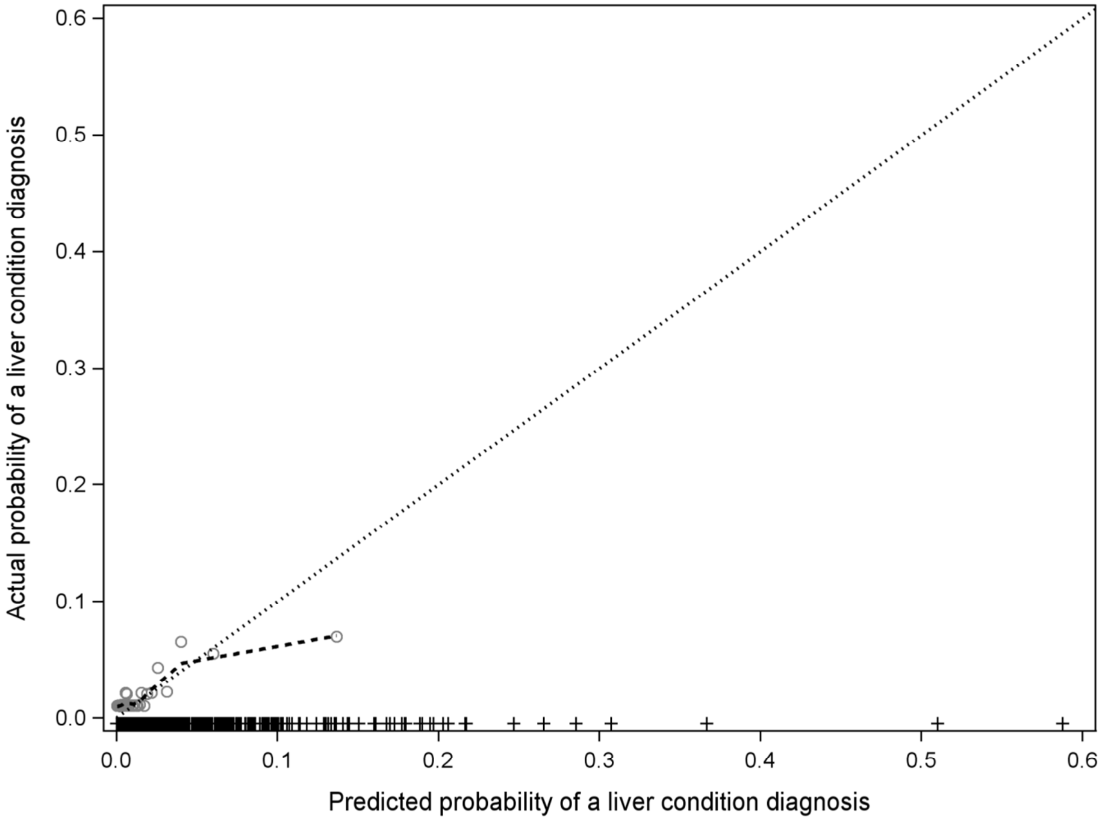

For peer review only - http://bmjopen.bmj.com/site/about/guidelines.xhtml

Note: Dashed line represents the smooth non-parametric Loess calibration curve; the circles represent the mean risks for subgroups of patients; the dotted line represents the perfect relationship; the plus symbols represent the spread of patients across predicted risks.

For peer review only

Appendix 5: Calculating the risk of liver disease diagnosis within two years from the final model.

Predictor	Description	Possible values
Gender	Male or female	=1 if Male<br>=0 if Female
Age	Age	16 to 103
Deprived	Deprivation based on Carstairs category linked to postcode	=1 if Yes (Carstairs categories 4 to 7)<br>=0 if No (Carstairs categories 1 to 3)
Alcohol	Previous hospital admission for alcohol dependency	=1 if Yes<br>=0 if No
Methadone	Ever prescribed methadone	=1 if Yes<br>=0 if No
ALP	Alkaline phosphatase result	9U/L to ∞
GGT	Gamma-glutamyltransferase result	2U/L to ∞
Albumin	Albumin result	14 to 63g/L
Transaminase	Alanine transaminase/aspartate aminotransferase result	4U/L to ∞

First calculate the linear predictor, $X\beta$, using the possible values contained within the table above:

$X\beta$=9.5239 – ***A*** + ***B***

where:

A= 0.5179*Deprived + 1.2097*Alcohol + 0.7385*LOG_e(ALP) + 1.7036*LOG_e(GGT+0.5) + 0.0700*Albumin*LOG_e(Transaminase) + 0.2954*Methadone*Albumin

and

B= 0.5827*Gender + 0.0138*Age + 8.3192*Methadone + 2.0162*LOG_e(Transaminase) + 0.4876*Albumin

Now, calculate Z:

$$Z = \frac{LOG_e(T) - X\beta}{4.5509}$$

Where ***T***= follow-up time, choose from 1 to 730 days

Predicted risk of liver disease within *T* days = Φ(***Z***),

i.e. the probability of Z from the standard Normal distribution. This can be found using a software package such as Microsoft Excel. In Excel use the following function: NORMSDIST(***Z***).

For peer review only

A worked example

Suppose we want to calculate the risk of liver disease within 2 years for a 34 year-old male methadone user living in a deprived area. He has the following LFT results: ALP=132U/L, albumin=40g/L, GGT=150U/L and Transaminase=63U/L

Xβ=9.5239 – ***A*** + ***B***

where:

A= 0.5179*1 + 1.2097*0 + 0.7385*LOG_e(132) + 1.7036*LOG_e(150.5) + 0.0700*40*LOG_e(63) + 0.2954*1*40

and

B= 0.5827*1 + 0.0138*34 + 8.3192*1 + 2.0162*LOG_e(63) + 0.4876*40

Xβ=9.5239 – 36.0824 + 37.2285

=10.67

$$Z = \frac{\text{LOG}(730) - 10.67}{4.5509}$$

= -0.8959

Predicted risk of liver disease within 730 days = Φ(**-0.8959**) = **0.1852** using the function NORMSDIST(Z) in Excel (i.e. NORMSDIST(-0.8959)).

For peer review only

Appendix 6 Scatterplot of the predicted probability of liver disease diagnosis from the full model against the predicted probability from the scoring tool.

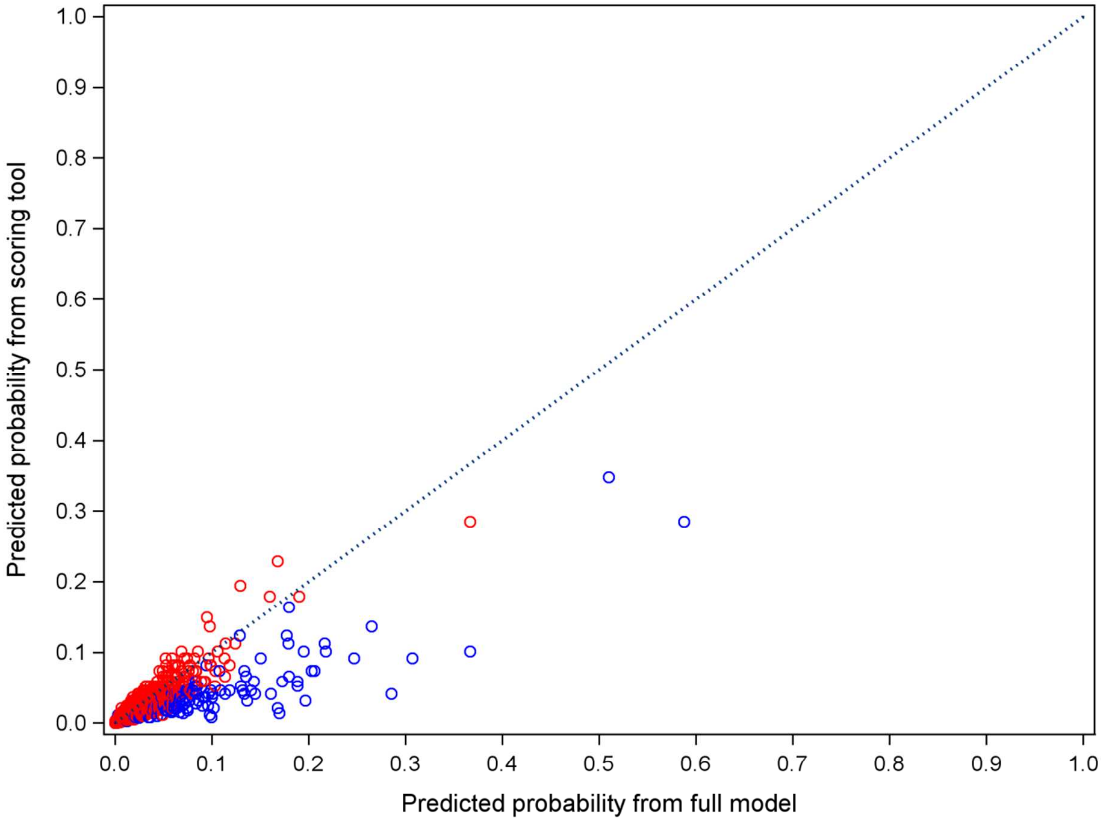

Note: The blue circles represent LFT values that would most definitely provoke referral from the GP to illustrate how the tool under-estimates the predicted probability of liver disease diagnosis for very high LFTs. In such circumstances the GP would automatically refer without the need for a scoring tool. The red circles are therefore LFTs that are less than the blue circle LFT cut-offs. The blue circles are defined as follows: transaminase > 200U/L; alkaline phosphatase >400U/L; gamma-glutamyl transferase >300; or albumin <24g/L.

TITLE: Prediction of liver disease diagnosis in primary care using the Algorithm for Liver Function Investigations (ALFI): model development and validation
Supplementary file

STROBE Statement—Checklist of items that should be included in reports of ***cohort studies***

	Item No	Recommendation	Location within article
**Title and abstract**	1	(*a*) Indicate the study's design with a commonly used term in the title or the abstract	Page 1 (title)<br>Page 2 (abstract)
		(*b*) Provide in the abstract an informative and balanced summary of what was done and what was found	Page 2
**Introduction**			
Background/rationale	2	Explain the scientific background and rationale for the investigation being reported	Page 4
Objectives	3	State specific objectives, including any prespecified hypotheses	Page 4-5
**Methods**			
Study design	4	Present key elements of study design early in the paper	Pages 7 and 8
Setting	5	Describe the setting, locations, and relevant dates, including periods of recruitment, exposure, follow-up, and data collection	Pages 7 and 8 (Derivation cohort)
Participants	6	(*a*) Give the eligibility criteria, and the sources and methods of selection of participants. Describe methods of follow-up	Pages 5 and 6
		(*b*) For matched studies, give matching criteria and number of exposed and unexposed	n/a
Variables	7	Clearly define all outcomes, exposures, predictors, potential confounders, and effect modifiers. Give diagnostic criteria, if applicable	Page 7 (Baseline characteristics & Outcomes)
Data sources/ measurement	8*	For each variable of interest, give sources of data and details of methods of assessment (measurement). Describe comparability of assessment methods if there is more than one group	Pages 6 and 21 (Databases and Box 1)
Bias	9	Describe any efforts to address potential sources of bias	Pages 8 and 9 (Model derivation and validation)
Study size	10	Explain how the study size was arrived at	n/a (whole population)
Quantitative variables	11	Explain how quantitative variables were handled in the analyses. If applicable, describe which groupings were chosen and why	Pages 7 and 8 (Baseline characteristics, Outcomes, Model derivation)
Statistical methods	12	(*a*) Describe all statistical methods, including those used to control for confounding	Pages 7-9<br>Further detail in web appendix (methods section p1-3)
		(*b*) Describe any methods used to examine subgroups and interactions	Page 8 (Model derivation)
		(*c*) Explain how missing data were addressed	Page 8 (Model derivation)

For peer review only - http://bmjopen.bmj.com/site/about/guidelines.xhtml

		(*d*) If applicable, explain how loss to follow-up was addressed	Page 7-8 (Model derivation)
		(*e*) Describe any sensitivity analyses	Page 9 (Model validation)
**Results**			
Participants	13*	(a) Report numbers of individuals at each stage of study—eg numbers potentially eligible, examined for eligibility, confirmed eligible, included in the study, completing follow-up, and analysed	Page 10
		(b) Give reasons for non-participation at each stage	As above
		(c) Consider use of a flow diagram	n/a
Descriptive data	14*	(a) Give characteristics of study participants (eg demographic, clinical, social) and information on exposures and potential confounders	Pages 10 and 22 (Baseline characteristics and Table 1)
		(b) Indicate number of participants with missing data for each variable of interest	Page 10
		(c) Summarise follow-up time (eg, average and total amount)	Page 10 (Liver disease diagnosis)
Outcome data	15*	Report numbers of outcome events or summary measures over time	Page 10 (Liver disease diagnosis)
Main results	16	(*a*) Give unadjusted estimates and, if applicable, confounder-adjusted estimates and their precision (eg, 95% confidence interval). Make clear which confounders were adjusted for and why they were included	Pages 11-12, 24 (only adjusted models presented)
		(*b*) Report category boundaries when continuous variables were categorized	Page 10 (Baseline characteristics) & Page 23 and 24 (footnotes to Table 1 and 2)
		(*c*) If relevant, consider translating estimates of relative risk into absolute risk for a meaningful time period	n/a
Other analyses	17	Report other analyses done—eg analyses of subgroups and interactions, and sensitivity analyses	Page 12 (validation) & web appendix (Appendices 2 and 3)
**Discussion**			
Key results	18	Summarise key results with reference to study objectives	Page 13
Limitations	19	Discuss limitations of the study, taking into account sources of potential bias or imprecision. Discuss both direction and magnitude of any potential bias	Pages 13-14
Interpretation	20	Give a cautious overall interpretation of results considering objectives, limitations, multiplicity of analyses, results from similar studies, and other relevant evidence	Pages 14-15
Generalisability	21	Discuss the generalisability (external validity) of the study results	Pages 14-15 (External validation text) & web appendices 2 and 3.

For peer review only

For peer review only - http://bmjopen.bmj.com/site/about/guidelines.xhtml

Other information			
Funding	22	Give the source of funding and the role of the funders for the present study and, if applicable, for the original study on which the present article is based	Page 16

*Give information separately for exposed and unexposed groups.

Note: An Explanation and Elaboration article discusses each checklist item and gives methodological background and published examples of transparent reporting. The STROBE checklist is best used in conjunction with this article (freely available on the Web sites of PLoS Medicine at http://www.plosmedicine.org/, Annals of Internal Medicine at http://www.annals.org/, and Epidemiology at http://www.epidem.com/). Information on the STROBE Initiative is available at http://www.strobe-statement.org.

For peer review only
